# Uropathogenic *Escherichia coli* infection-induced epithelial trained immunity impacts urinary tract disease outcome

**DOI:** 10.1038/s41564-023-01346-6

**Published:** 2023-04-10

**Authors:** Seongmi K. Russell, Jessica K. Harrison, Benjamin S. Olson, Hyung Joo Lee, Valerie P. O’Brien, Xiaoyun Xing, Jonathan Livny, Lu Yu, Elisha D. O. Roberson, Rajdeep Bomjan, Changxu Fan, Marina Sha, Shady Estfanous, Amal O. Amer, Marco Colonna, Thaddeus S. Stappenbeck, Ting Wang, Thomas J. Hannan, Scott J. Hultgren

**Affiliations:** 1 Department of Molecular Microbiology and Center for Women’s Infectious Disease Research, Washington University School of Medicine, St Louis, MO USA; 2 Department of Genetics, Washington University School of Medicine, St Louis, MO USA; 3 Edison Family Center for Genome Sciences and Systems Biology, Washington University School of Medicine, St Louis, MO USA; 4 Fred Hutchinson Cancer Center, Human Biology Division, Seattle, WA USA; 5 Infectious Disease and Microbiome Program, The Broad Institute of Massachusetts Institute of Technology and Harvard, Cambridge, MA USA; 6 Department of Medicine, Division of Rheumatology, Washington University School of Medicine, St Louis, MO USA; 7 Department of Microbial Infection and Immunity, Infectious Diseases Institute, Ohio State University, Columbus, OH USA; 8 Biochemistry and Molecular Biology Department, Faculty of Pharmacy Helwan University, Cairo, Egypt; 9 Department of Pathology and Immunology, Washington University School of Medicine, St Louis, MO USA; 10 Department of Inflammation and Immunity, Lerner Research Institute, Cleveland Clinic, Cleveland, OH USA

**Keywords:** Bacterial infection, Mucosal immunology, Imprinting

## Abstract

Previous urinary tract infections (UTIs) can predispose one to future infections; however, the underlying mechanisms affecting recurrence are poorly understood. We previously found that UTIs in mice cause differential bladder epithelial (urothelial) remodelling, depending on disease outcome, that impacts susceptibility to recurrent UTI. Here we compared urothelial stem cell (USC) lines isolated from mice with a history of either resolved or chronic uropathogenic *Escherichia coli* (UPEC) infection, elucidating evidence of molecular imprinting that involved epigenetic changes, including differences in chromatin accessibility, DNA methylation and histone modification. Epigenetic marks in USCs from chronically infected mice enhanced caspase-1-mediated cell death upon UPEC infection, promoting bacterial clearance. Increased *Ptgs2os2* expression also occurred, potentially contributing to sustained cyclooxygenase-2 expression, bladder inflammation and mucosal wounding—responses associated with severe recurrent cystitis. Thus, UPEC infection acts as an epi-mutagen reprogramming the urothelial epigenome, leading to urothelial-intrinsic remodelling and training of the innate response to subsequent infection.

## Abstract (web summary)

Uropathogenic *Escherichia coli* infection induces epigenetic remodelling and trained immune responses in bladder epithelial stem cells that affect susceptibility to recurrent infection.

## Main

Urinary tract infections (UTIs) are one of the most common bacterial infections worldwide and are a substantial cause of morbidity in otherwise healthy females^1,2^. The high recurrence rate in susceptible individuals makes treatment challenging^3^, with one of the strongest risk factors for developing a UTI being a previous UTI^2^. The biological basis for recurrent UTI (rUTI) is poorly understood.

Without antibiotic treatment, acute UTIs in humans either self-resolve or develop into long-lasting chronic infections^4^. Infection of C3H/HeN mice with uropathogenic *Escherichia coli* (UPEC) recapitulates these two outcomes. Chronic cystitis in these mice is defined as persistent high titre bacteriuria (bacteria in urine) accompanied by chronic inflammation (sensitized mice), while resolution of infection defines resolved mice^5^. Analysis of bladders from resolved and sensitized mice reveals that infection leads to differential bladder remodelling that impacts susceptibility to rUTI. Resolved mice are resistant to rUTI, in part because they have an accelerated, transient bladder TNFα/cyclooxygenase-2 (Cox-2) response, which promotes rapid elimination of infection and mucosal healing^6,7,8^. Sensitized mice are highly susceptible to rUTIs upon challenge, with robust sustained bladder TNFα and Cox-2 expression causing neutrophil transmigration across the bladder epithelium (urothelium) and mucosal wounding that promotes severe recurrent bacterial infections^6,7,8^. Thus, depending on disease history, the bladder tissue is differentially remodelled in a way that either increases or decreases susceptibility to rUTI.

These phenotypes led to our hypothesis that bladder mucosal remodelling is mediated in part by epigenetic changes in urothelial stem cells (USCs) that impact bladder mucosal defence against subsequent infections^6,7^. Thus, we isolated epithelial cells from the bladders of mice with different disease histories, established primary USC lines, propagated them in cell culture, and formed polarized and fully differentiated urothelium in vitro, which displayed morphological phenotypes that resembled the urothelial remodelling phenotypes observed in vivo^6^. We identified differences in chromatin accessibility, DNA methylation and histone modifications in the USC lines, which corresponded with differences in transcriptional responses affecting programmed cell death and cyclooxygenase-2 (Cox-2)-regulated inflammatory responses that impact rUTI susceptibility. Overall, our study provides epigenetic evidence of epithelial-intrinsic trained immunity caused by a mucosal bacterial infection, which alters the bladder mucosal response to subsequent infections, depending on the original disease outcome. This finding may explain the high prevalence of rUTI and have important therapeutic implications for chronic/recurrent bacterial infections in general.

## Results

### Differentiated urothelial cells form urothelial barriers

**Fig. 1:**
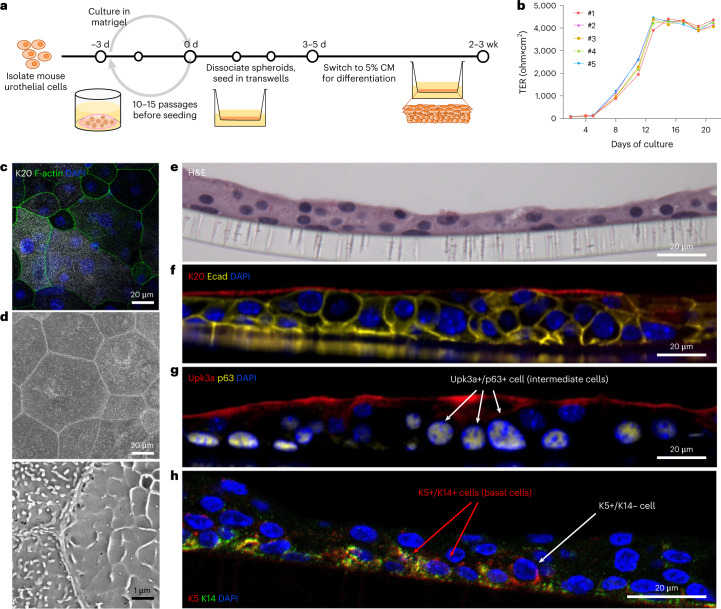
The culture of USCs of juvenile C3H/HeN mice regenerates differentiated urothelium in vitro. **a**, USCs isolated from 8-week-old C3H/HeN mice were expanded by spheroidal culture in matrigel with 50% L-WRN conditioned media (CM) including Y-27632, a ROCK inhibitor, and SB431542, a TGF β type 1 inhibitor. After 3 d of spheroid culture, cells were dissociated into a single-cell suspension and 3–4 × 10^5^ cells were seeded onto transwell membranes. The cells were cultured in 50% CM for 3–5 d, then cultured in 5% CM for 2–3 weeks until full differentiation. **b**, Cell cultures with a TER value >4,000 ohm × cm^2^ were then analysed (5 transwells cultured from one juvenile C3H/HeN cell line). For consistency, TER was measured 1 d after media change. **c**,**d**, Differentiated urothelia on the transwells were fixed and imaged via (**c**) confocal microscopy and (**d**) SEM to show a top-down view of the urothelium at magnification 500× (top panel) and 10,000× (bottom panel). In **c**, samples were stained for F-actin, the terminal differentiation marker K20 and nuclei (DAPI). **e**–**h**, The urothelia were also paraffin-embedded, sectioned and stained with hematoxylin and eosin (H&E) (**e**) and immunostained for K20, Ecad and DAPI (**f**), Upk3a, p63 and DAPI (**g**) or K5, K14 and DAPI (**h**). Representative images are shown. Data are from 2–3 independent experiments using USCs from 5 different juvenile C3H/HeN mice. Source data

**Extended Data Fig. 1:**
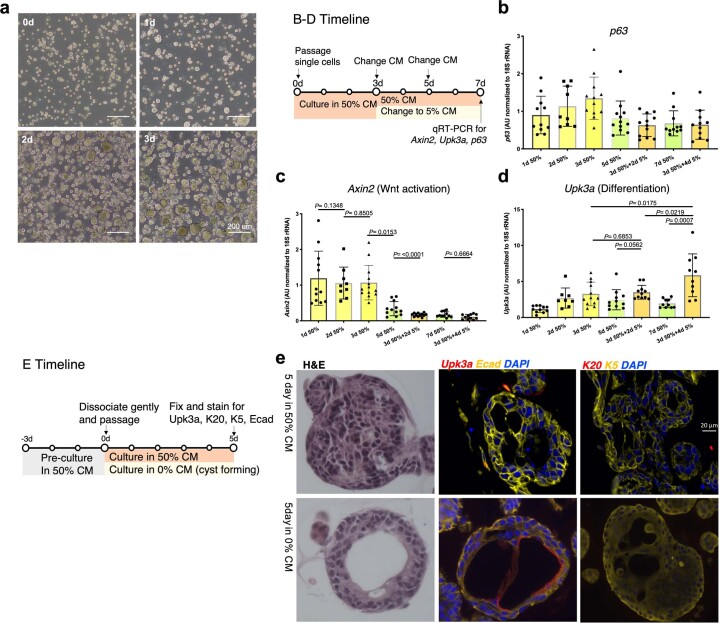
Primary epithelial stem cells possess differentiation potential and stemness, related to Fig. 1. **(A)** For the cell expansion, dissociated cell aggregates were embedded in fresh matrigel then developed into new spheroids. Urothelial spheroids can be passaged every 3 days using 1:2–1:3 dilutions depending on cell density. **(B-D)** Primary USCs originated from 8 week old C3H/HeN mice were cultured in matrigel with 50% CM. After 3 days of culture in 50% CM, media were changed to fresh 50% CM or 5% CM at 3, 5, and 7 days, then RNAs were isolated at 1, 2, 3 (yellow), 5 (green), and 7 days (orange) (USC culture n = 12, 9, 13, 12, 12, 11, 11 respectively). **(B)** Gene expression of *p63*, **(C)** *Axin2*, a Wnt signaling marker, and **(D)** *Upk3a*, a urothelial cell differentiation marker, was measured by qRT-PCR and data is represented as mean ± SD. Significance was determined by an unpaired (two-tailed) t test. **(E)** To culture bladder organoids in matrigel, USCs were pre-cultured in 50% CM for 3 days, gently dissociated, then passaged into fresh matrigel for culture in 50% CM or 0% CM for 5 days, while media were changed every 2 days. After 5 day culture, USC spheroids were fixed with 10% neutral buffered formaldehyde (NBF) and prepared for paraffin embedding. The slide with paraffin sections were stained with hematoxylin and eosin (H&E) and immunostained for Upk3a (red), E-cadherin (yellow), and DAPI (blue) or K20 (red), K5 (yellow), and DAPI (blue). Source data

To study urothelial-intrinsic changes that result from previous infection, we adapted a method for in vitro propagation of primary intestinal epithelial stem cells in three-dimensional (3D) culture^9,10^ to culture primary USCs that were isolated from 8-week-old naïve C3H/HeN mice (Fig. 1a and Extended Data Fig. 1a). Gene expression of *p63*, which encodes transformation-related protein 63 (p63), was measured because it is essential for the proliferative capacity of the stem cells^11^, while the expression of *Axin2*, which is a Wnt-target gene^10^, was measured to assess Wnt signalling activity in the 3D culture. Together, we can evaluate the stem cell pluripotency status by monitoring expression of these genes with RT–qPCR (Extended Data Fig. 1b,c). *p63* and *Axin2* gene expression remained high through the first 3 d of 3D culture in 50% percent conditioned media (CM), which is required to maintain pluripotency. Expression of *p63* and *Axin2* then decreased at 5 to 7 d post-infection, regardless of whether the cells were incubated in 50% or 5% CM at day 3 post-infection, showing that either extended culture or lower CM percentage can reduce Wnt signalling. However, uroplakin 3a (Upk3a), a surface protein expressed by differentiated urothelial cells, was only significantly increased in cells grown in 5% CM but not in 50% CM, as determined at 7 d post-infection (Extended Data Fig. 1d). After propagation of USCs in 50% CM over several passages and further culturing in 50% CM over 5 d, all cells stained positive for the epithelial cell marker E-cadherin and the basal urothelial cell marker keratin 5 (K5) but lacked Upk3a expression (Extended Data Fig. 1e). In contrast, culturing in 0% CM in 3D culture for 5 d after initial propagation resulted in the development of epithelial polarity with the formation of a central cavity (Extended Data Fig. 1e). Within the resulting cysts, cavity-facing cells differentiated into superficial facet-like cells (Upk3a+, K5−), whereas perimeter cells in contact with the matrigel matrix were basal cell-like (Upk3a–, K5+).

**Extended Data Fig. 2:**
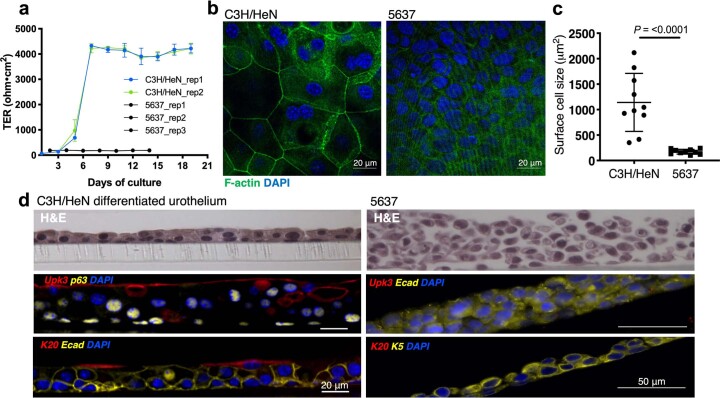
The differentiated urothelium better recapitulates bladder tissue phenotypes than the human bladder carcinoma cell line 5637 does, related to Fig. 1. **(A)** Primary C3H/HeN urothelial cells and 5637 cells were cultured in Transwells for 2–3 weeks and transepithelial electrical resistance (TER) of Transwells were measured every 2 days before media change. Data collected from each cell lines (n = 3 for each) represented as mean ± SD. **(B)** Whole mount urothelium of both cell types were fixed and stained for confocal microscopy analysis; F-actin (green) and DAPI (blue). **(C)** Surface cell size of primary C3H/HeN urothelial cells and 5637 cells was measured using confocal images (n = 10 each). Data are represented as mean ± SD and significance was determined by an unpaired (two-tailed) t test (p-value <0.001). **(D)** The Transwell cultures of both C3H/HeN and 5637 cells were fixed, cut into slices, and then processed for paraffin embedding. Histologic sections were cut and stained with H&E or immunostained for Upk3a, K20, Ecad, K5, p63, and DAPI. Source data

We next established a transwell culture system to differentiate USCs of juvenile C3H/HeN mice into polarized, stratified urothelial barriers (Fig. 1a). The 5637 human bladder carcinoma cell line (ATCC HTB-9), which has been used widely for the in vitro study of UPEC interactions with bladder cells and originates from basal bladder cancer cells^12^, was used as a control. As both 5637 cells and our primary USCs are of basal cell origin, we seeded and cultured them on transwells for 2–3 weeks to compare cell differentiation phenotypes. Formation of intact differentiated urothelium, referred to herein as differentiated urothelium (or urothelia), by the primary USCs was confirmed by robust transepithelial electrical resistance (TER) (Fig. 1b), strong confocal staining of large hexagonal superficial facet cells for the terminal differentiation marker K20 (Fig. 1c), and the presence of cell junctions and uroplakin plaques on the tissue surface (Fig. 1d), similar to how superficial facet cells appear on the surface of intact bladder tissues^6^. Microscopic analysis of differentiated urothelial sections revealed a multilayer polarized epithelial (Ecad+) tissue layer (Fig. 1e–h), with p63, K5 and K14 expression in the basal layer cells above the matrigel membrane (mostly K5+/K14+, a few K5+/K14−), p63 and weak Upk3a expression in some midlayer cells, indicative of intermediate cells, and strong K20 and Upk3a staining on the surface of the apical layer cells, indicating terminally differentiated superficial facet cells. Altogether, the distribution of these differentiation characteristics in basal, intermediate and superficial urothelial layers is consistent with that observed in mouse and human bladder tissue^13^. In contrast, none of these features were observed in 5637 cell transwell cultures, in which the cells were loosely packed together in layers 4–6 cells deep, but lacked evidence of polarization or cell junction formation (Extended Data Fig. 2a–d). Thus, primary USCs provide important advantages over a tumour cell line for studying the urothelium.

### Differentiated urothelia mimic in vivo infection phenotypes

**Fig. 2:**
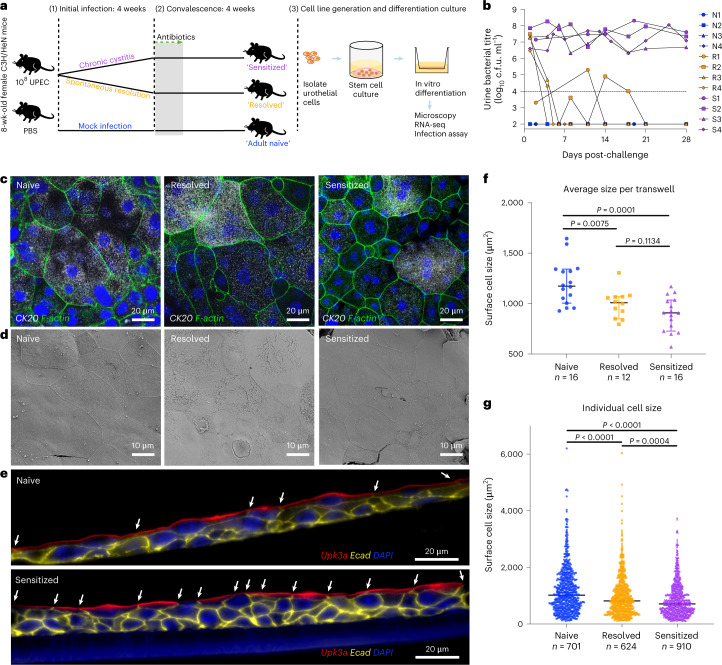
Differentiated urothelia originating from previously infected mice maintain bladder remodelling phenotypes. **a**, Time course of initial infection with 10^8^ c.f.u. UTI89 Kan^R^ and convalescent period in C3H/HeN mice. **b**, Representative urine bacterial titre time course over 4 wpi. Horizontal line represents the cut-off for notable bacteriuria: 10^4^ c.f.u. ml^−1^. Naïve, resolved and sensitized mice were named as N1-4, R1-4 and S1-4. **c**,**d**, USCs isolated from these mice were cultured into differentiated urothelia on transwells, fixed and imaged via confocal microscopy (**c**) and SEM (**d**). In **c**, the urothelia were stained for K20, F-actin (Phalloidin) and nuclei (DAPI). **e**, Transwells were paraffin-embedded, sectioned and immunostained for Upk3a, E-cadherin and nuclei. White arrows show cell junctions indicating size of surface cells. **f**,**g**, Fixed slides processed from 44 transwells of naïve, resolved and sensitized mice (*n* = 16, 12 and 16 transwells from *n* = 4, 3, 4 mice, respectively) were stained for K20, E-cadherin and nuclei, labelled and imaged in a double-blind manner. Then the superficial cell sizes were automatically measured using the Fiji ImageJ macro programme and plotted for average cell size per transwell (**f**) and individual cell size (**g**), represented as median with 95% CI. Two-tailed Student’s *t*-test was used to determine significance and *P* values are indicated when significant. Source data

Using our murine model of rUTI^5,6^, we have shown that an initial UTI event results in long-lasting bladder remodelling, including structural and proteomic changes to the urothelium, that impact susceptibility to rUTI^14^. To investigate the role of USCs in bladder remodelling, we isolated bladder USCs from convalescent mice (4 weeks after the initiation of antibiotics), from both those with self-resolved infection (resolved) and those that developed chronic infection (sensitized), as well as from age-matched naïve mice as controls and established USC lines (*n* = 4 per infection history) (Fig. 2a,b). We propagated each of these USC lines for between 15 and 30 passages, then differentiated them on transwells and characterized each cell line by microscopy after 2–3 weeks of culture. Strikingly, we found that the urothelium derived from sensitized USCs recapitulated many of the morphological differences observed previously in vivo^6^ even after many passages, including smaller surface cells and decreased expression of the terminal differentiation markers Upk3a and K20 when compared with differentiated urothelium derived from naïve and resolved USCs (Fig. 2c–e). Automated measurement of surface cell sizes showed that the apical cells of the sensitized differentiated urothelia are significantly smaller than those in naïve differentiated urothelia, whereas the resolved urothelia have an intermediate phenotype, consistent with in vivo data (Fig. 2f–g)^6^. Collectively, these data indicate that transwell culture of USCs can recapitulate the morphological bladder epithelial remodelling phenotypes seen in vivo.

### Epigenetic characteristics associate with disease history

**Fig. 3:**
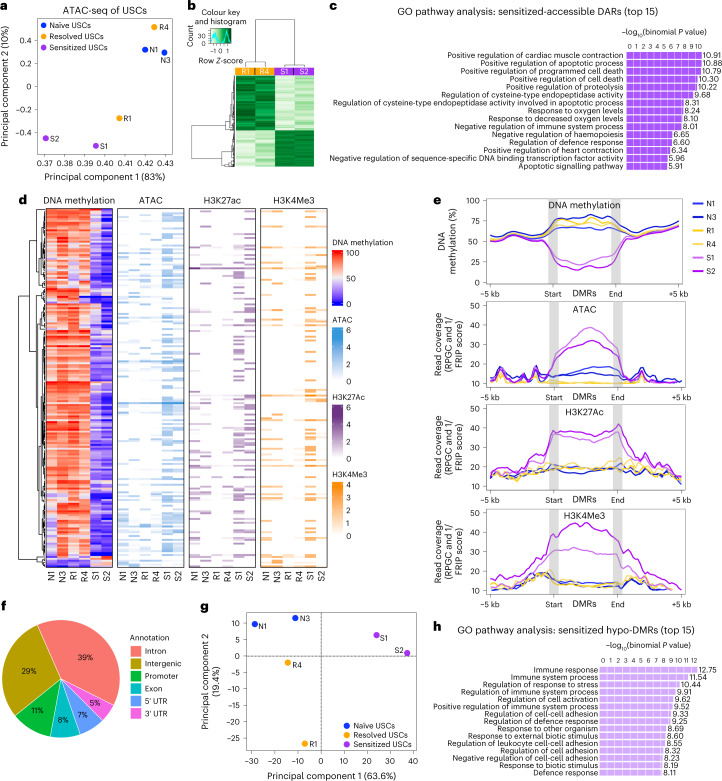
Convalescent mouse USCs have differential epigenetic memories upon UPEC infection. Omni-ATAC-seq was performed using USCs from naïve, resolved and sensitized mice (cell lines N1, N3, R1, R4, S1 and S2, each from an individual mouse). **a**, PCA plot of DARs across the USC lines. **b**, Heat map of significantly differential peaks (FDR < 0.05) comparing sensitized vs resolved USCs. Out of all 2,880 DARs, 925 regions are sensitized-accessible DARs and 1,955 regions are resolved-accessible DARs. **c**, The top 15 enriched GO terms for sensitized-accessible DARs (*n* = 747, fold change >1.5, FDR < 0.05) were analysed using GREAT. Significance was determined using the binomial test. **d**,**e**, Differences in chromatin accessibility, DNA methylation and active histone modifications, H3K27Ac and H3K4Me3, in different USCs were assessed by ATAC-seq, whole genome bisulfite sequencing (WGBS) and CUT&RUN. **d**, Sensitized-specific DMRs (*n* = 189) among naïve, resolved and sensitized USCs are visualized as a series of heat maps displaying the epigenetic landscape overlapping each DMR for DNA methylation, ATAC and active histone modifications H3K27Ac and H3K4Me3. **e**, Average signals 5 kb upstream and 5 kb downstream of sensitized-specific hypo-DMRs (*n* = 183) for WGBS, ATAC, H3K27Ac and H3K4Me3 are visualized for each cell line. Average length of DMRs is 362 base pairs. DMRs are scaled for size with ‘start’ and ‘end’ of DMRs, which are indicated as grey bars. **f**, Sensitized-specific hypo-DMRs were annotated according to their predicted locations in the genome (*n* = 183). **g**, A PCA plot of all DMR comparisons showing clustering of the different cell lines. **h**, The top 15 enriched GO terms for sensitized hypo-DMRs were analysed using GREAT (*n* = 183). Source data

Our data indicate that a previous infection results in USC-intrinsic changes that are heritable over many generations of cell culture. Therefore, we hypothesized that these changes are mediated by differential epigenetic modification in the USCs that associate with: (1) disease history and (2) genome-wide differences in chromatin accessibility. Chromatin accessibility was measured by Omni-ATAC-seq, a technique for sequencing regions of nuclear chromatin that are accessible to a transposase by sequencing^15^. We identified a total of 59,801, 63,195 and 82,030 highly reproducible accessible chromatin regions in two biological replicates each of the naïve, resolved and sensitized USC lines, respectively. Principal component analysis (PCA) of these USC lines separates sensitized USCs from other groups (Fig. 3a). Among 2,880 differentially accessible regions (DARs) of chromatin identified between sensitized and resolved USCs, 925 regions are sensitized-accessible DARs (more accessible in sensitized than in resolved) and 1,955 regions are resolved-accessible DARs (more accessible in resolved than in sensitized) (Fig. 3b).

**Extended Data Fig. 3:**
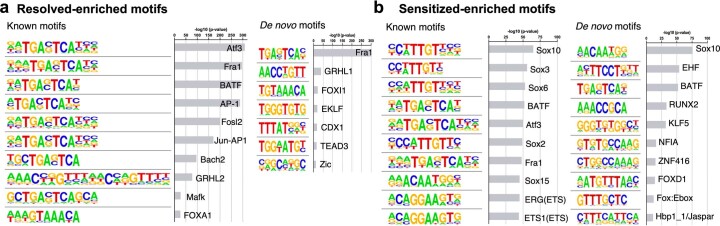
Enriched motifs of Sensitized and Resolved-specific DARs, related to Fig. 3 and Fig. 5. **(A-B)** HOMER motif analysis using USC ATAC-seq data (Fig. 4a) generated lists of enriched TF binding motifs in Sensitized-accessible DARs **(A)** and Resolved-accessible DARs **(B)** with their p-values. HOMER scanned the sequences of DARs for known motifs and calculate enrichment score p-value using binomial test. HOMER also discovered *de novo* motifs with their best matches to a known motif in DARs. Each Top 10 known and *de novo* motifs enriched in Sensitized **(A)** and Resolved USCs **(B)** are shown with their sequence logo and p-value.

Gene Ontology (GO) pathway analysis on DARs using GREAT^16^ revealed that genes associated with sensitized-accessible DARs are strongly enriched for many biological processes, including those involving programmed cell death (PCD), oxidative stress and immune response (Fig. 3c). Many of these DARs were in proximity to PCD pathway associated genes, including *Casp1* and *Gsdmc2/3* (Supplementary Table 1), which were previously found to be enriched in both whole-bladder RNA-seq and ex vivo urothelial proteomic comparisons of sensitized vs resolved mice^6,8^. Performing motif discovery analysis using HOMER^17^, we found that resolved-accessible DARs are highly enriched for transcription factor (TF) binding motifs of the AP-1 family members, which are known mediators of stress responses, often downstream of cytokine signalling (Extended Data Fig. 3a). In contrast, sensitized-accessible DARs are enriched not only for TF binding motifs of the AP-1 family, but also for those that regulate stem cell fate and tissue differentiation and development, including SOX family members EHF, Klf5 and RUNX2 (Extended Data Fig. 3b).

**Extended Data Fig. 4:**
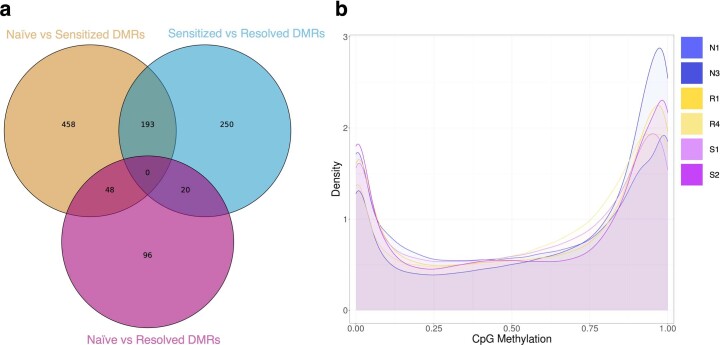
DNA methylation distribution for cell lines and cell line-specific differentially methylated regions (DMRs). **(A)** Venn diagram of all the DMR comparisons with the numbers of each of the comparisons. The Sensitized-specific DMRs are shown in the overlap between Naïve vs Sensitized and Sensitized vs Resolved DMR comparisons. **(B)** The density of CpG methylation shows a bimodal distribution with no global differences in DNA methylation between the three groups (Naïve, Resolved and Sensitized USCs), where CpG methylation represents the fraction of total reads that are methylated per CpG site. Source data

**Extended Data Fig. 5:**
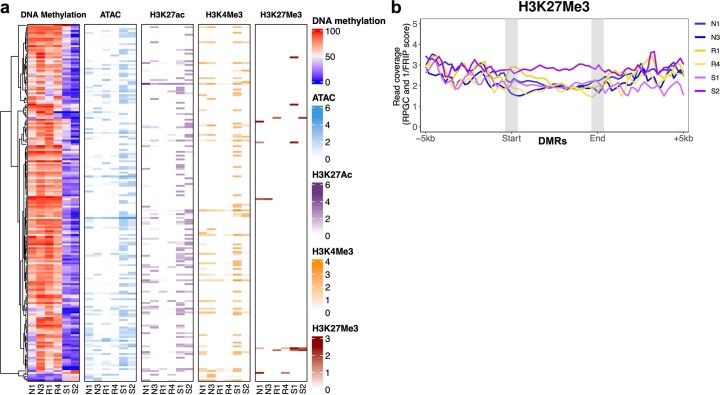
The repressive histone mark H3K27Me3 is not different between Naïve, Resolved, and Sensitized cell types, related to Fig. 3. **(A)** Sensitized-specific DMRs among Naïve, Resolved, and Sensitized USCs are visualized as a series of heatmaps displaying DNA methylation, ATAC, and histone modifications: H3K27Ac (active promoter/enhancer), HeK4Me3 (promoter), and H3K27Me3 (polycomb repression). **(B)** Average signals 5 kb upstream and 5 kb downstream of Sensitized-specific hypo-DMRs for H3K27Me3 (polycomb repression) are visualized for each cell line.

Chromatin remodelling is accomplished through two main mechanisms: DNA methylation and histone modifications. We performed genome-wide profiling of DNA methylation and histone modification in our USC lines using WGBS^18^ and CUT&RUN^19^, respectively. For CUT&RUN, we selected antibodies against histone modification marks: H3 lysine 4 trimethylation (H3K4Me3), H3 lysine 27 acetylation (H3K27Ac) and H3 lysine 27 trimethylation (H3K27Me3), which are associated with active promoters, active promoter/enhancers and polycomb repression, respectively^20^. Most of the differentially methylated regions (DMRs) were identified in the comparisons of sensitized USCs with either (1) naïve or (2) resolved USCs. We did not observe any global differences in DNA methylation between naïve, resolved and sensitized groups (Extended Data Fig. 4a,b). Sensitized-specific DMRs are displayed as a heat map together with the ATAC-seq and CUT&RUN peaks to visualize the epigenetic landscape across these DMRs (Fig. 3d). We found that sensitized-specific DMRs tend to be hypo-methylated (hypo-DMR) compared with naïve and resolved USCs (Fig. 3d). DNA methylation can reduce gene expression by inhibiting transcription factor binding and potentially affecting nucleosome occupancy, together resulting in less accessible chromatin^21^. Concordantly, we found that hypo-DMRs in sensitized USCs have corresponding increased ATAC-seq, H3K4Me3 and H3K27Ac signals (Fig. 3d), suggesting that these regions are enriched in marks for active promoters and enhancers. Looking at the average signals in the genomic regions (+/− 5 kb) surrounding sensitized-specific hypo-DMRs, we saw similar patterns of hypo-methylation, increased chromatin accessibility and increased active histone marks compared with naïve and resolved USCs (Fig. 3e). However, the repressive histone modification, H3K27Me3, was not correlated with sensitized-specific DMRs (Extended Data Fig. 5a,b). The sensitized-specific hypo-DMRs are more associated with genic features such as promoters, exons and introns (Fig. 3f). A PCA plot of all DMRs separated sensitized USCs from naïve and resolved USCs (Fig. 3g). GO pathway analysis of sensitized-specific hypo-DMRs showed enrichment in immune response and cell-cell adhesion-related pathways (Fig. 3h). Thus, USCs maintain epigenetic memories of a previous UPEC infection that differ on the basis of the initial infection outcome and are mediated by differential DNA methylation and active histone modifications.

### USC differentiation programmes associate with disease history

**Fig. 4:**
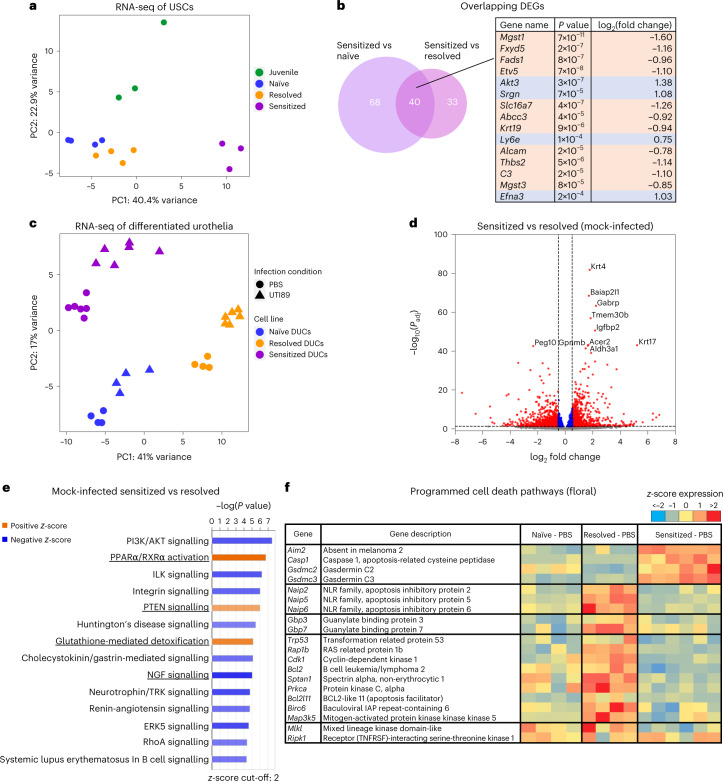
Differentiated urothelia originating from convalescent mice maintain differential transcriptomics observed in vivo. RNAs were isolated from (**a**,**b**) undifferentiated juvenile naïve, adult naïve, resolved and sensitized USCs (cell lines of *n* = 3, *n* = 4, *n* = 4, *n* = 3 from 14 mice), or (**c**–**f**) differentiated urothelia of naïve, resolved and sensitized cells (different cultures from N3, R3 and S3) with or without UPEC infection, then analysed by RNA-seq and by differential analysis. **a**, A PCA of USC RNA-seq by significantly differentially expressed genes shows that samples are clustered by cell lines (previous infection outcome). **b**, Forty differentially expressed genes (DEGs) were overlapping between sensitized vs naïve and sensitized vs resolved USCs. The top 15 overlapping DEGs are listed in the table. **c**, A PCA of differentiated urothelial RNA-seq shows clustering by cell lines (previous infection outcome) and secondary infection condition. **d**, A volcano plot comparing mock-infected sensitized vs resolved differentiated urothelia. DEGs with FC >0.5, *P*_adj_ <0.05 are indicated as red dots. **e**, Pathway analysis was used to assess the biological pathways enriched in differentially expressed genes in mock-infected sensitized relative to resolved differentiated urothelia. Significance was determined using a right-tailed Fisher’s exact test, with *P*_adj_ < 0.05 being considered as significantly enriched pathways. Shown are selected pathways with *z*-score >2 and –log(*P* value) >4.2 from the specific enriched pathways by IPA. Pathways overlapping between mock-infected and UPEC-infected differentiated urothelia (Extended data Fig. 8d) are underlined. **f**, A heat map showing programmed cell death associated genes that are differentially expressed in mock-infected naïve, resolved and sensitized differentiated urothelia. Significance of DEGs was determined using Wald test, followed by multiple test correction using Benjamini-Hochberg FDR for adjusted *P* value. Source data

**Extended Data Fig. 6:**
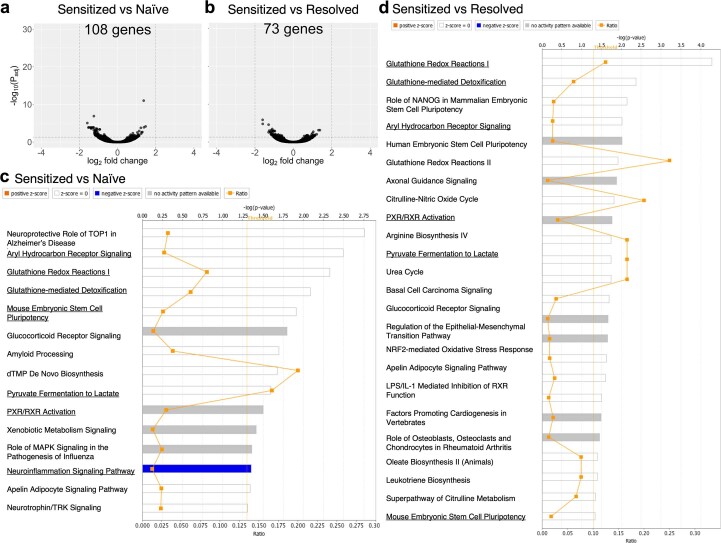
RNA-seq of primary USCs originating from convalescent mice revealed that sensitized USCs maintain differential gene expressions after several passages, related to Fig. 4. RNAs were isolated from Naïve, Resolved, and Sensitized USCs, then analysed by RNA-seq and performed differential analysis. Significance was determined by Wald test followed by multiple test correction using Benjamini-Hochberg FDR for adjusted p-value.108 and 73 genes were significantly differentially expressed in Sensitized USCs relative to **(A)** Naïve and **(B)** Resolved USCs (P-adj < 0.05). Enriched pathways in Sensitized USCs compared with **(C)** Naïve and **(D)** Resolved USCs are listed here. Overlapping pathways in both analyses are underlined. IPA determine significance using a right-tailed Fisher’s exact test, with P-adjusted <0.05 considered significantly enriched pathways. Source data

**Extended Data Fig. 7:**
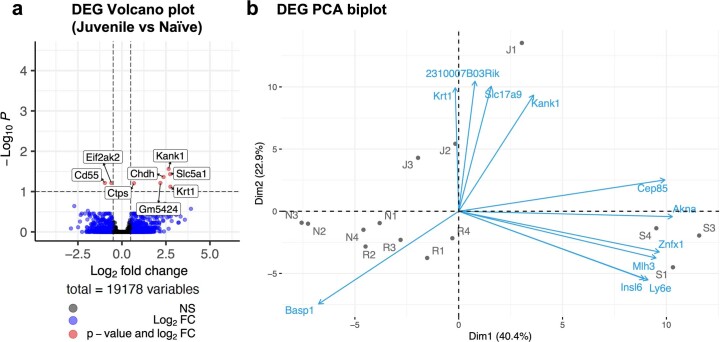
Juvenile Naïve USCs have a different gene expression profile with the other cell types, related to Fig. 4. RNA was isolated from Juvenile Naïve, Adult Naïve, Resolved and Sensitized USCs (cell lines of n = 3, n = 4, n = 4, n = 3 from 14 mice). Then, an RNA-seq analysis was performed as described in Fig. 4a. Significance was determined by Wald test followed by multiple test correction using Benjamini-Hochberg FDR for adjusted p-value. **(A)** A volcano plot of differentially expressed genes (DEGs) comparing Juvenile Naïve vs Adult Naïve USCs identify 8 significantly DEGs (FDR cutoff 0.1). **(B)** The PCA biplot for the PCA shown in Fig. 4a indicates how strongly each gene influences the principal components (PC). Genes including *Znfx1* and *Ly6e* strongly influence PC1 (Dim1) while genes including *Kank1* and *Krt1* strongly influence PC2 (Dim2). Source data

We then performed RNA-seq of naïve, resolved and sensitized USCs to investigate whether their altered epigenomes result in differential gene expression. We included juvenile naïve USCs (Fig. 1) as comparators for age differences. Again, PCA of all DEGs separates sensitized USCs from other groups along principal component (PC) 1 (Fig. 4a). Sensitized USCs had 108 and 73 differentially expressed genes (DEGs) compared with naïve and resolved USCs, respectively (Extended Data Fig. 6a,b), of which 40 genes were common to both comparisons (Fig. 4b). The top 15 DEGs included the glutathione transferase genes *Mgst1* and *Mgst3* (Fig. 4b). Enriched pathways in sensitized compared to naïve and resolved USCs include those related to protection against reactive oxidative species (ROS), nuclear receptor signalling and stem cell pluripotency (Extended Data Fig. 6c,d). In contrast, no genes were differentially expressed between resolved and naïve USCs. Juvenile naïve USCs separated from the adult naïve USCs mainly along PC2 (Fig. 4a), but this comparison only revealed 8 significant DEGs (Extended Data Fig. 7a). A PCA biplot shows that genes including *Znfx1* and *Ly6e* strongly influenced PC1, while genes including *Kank1* and *Krt1* strongly influenced PC2 (Extended Data Fig. 7b).

**Extended Data Fig. 8:**
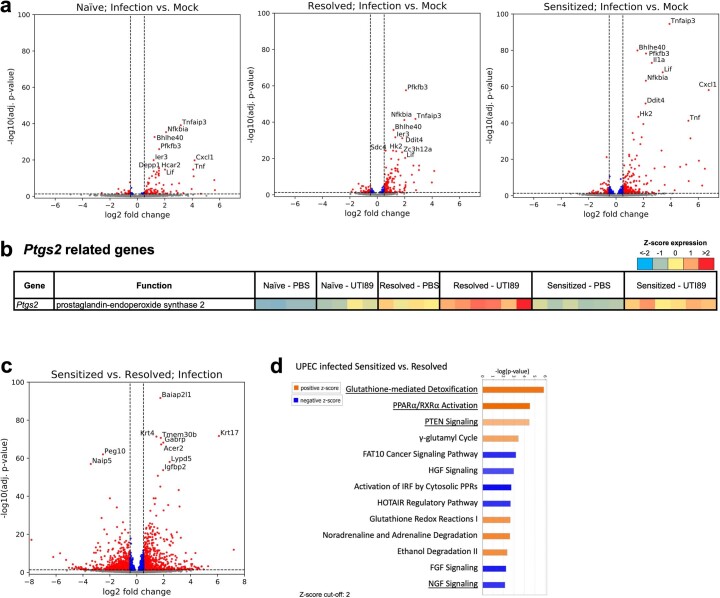
Differential transcriptomics of differentiated urothelia originating from convalescent mice recapitulate *in vivo* studies, related to Fig. 4. RNA-seq data of UTI89 infected and mock-infected differentiated urothelia (Fig. 4c) was used to perform differential analysis. **(A)** Volcano plots comparing UPEC infected vs mock-infected Naïve, Resolved, and Sensitized differentiated urothelia (UPEC infection response). **(B)** Differential gene expression of *Ptgs2* between Naïve, Resolved, and Sensitized differentiated urothelia with or without UTI89 infection is visualized as a heatmap. **(C)** A volcano plot comparing UTI89 infected Sensitized vs Resolved differentiated urothelia was performed. **(D)** Pathway analysis was used to assess the biological pathways enriched in differentially expressed genes in UPEC infected Sensitized differentiated urothelia relative to Resolved differentiated urothelia, and significance was determined by a right-tailed Fisher’s exact test, with P-adjusted <0.05 considered significantly enriched pathways. Shown are selected pathways with z-score > 2 and –log(p-value) > 2 from the specific enriched pathways by IPA. Source data

**Extended Data Fig. 9:**
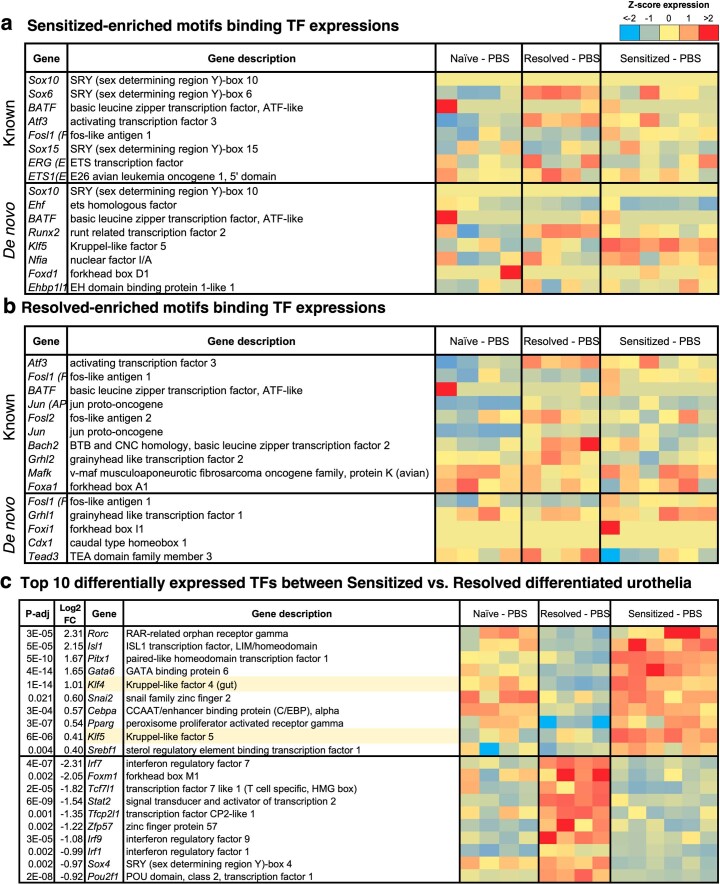
Differential gene expression of TFs between Sensitized vs. Resolved differentiated urothelia, related to Figs. 3 and 4. **(A-B)** HOMER motif analysis of ATAC-seq data in Fig. 4 was performed to show differential TF binding motif enrichment in the DARs found in Sensitized and Resolved USCs. Using a list of each top 10 known and *de novo* motifs of Sensitized-accessible DARs **(A)** and Resolved-accessible DARs **(B)** (Extended Data Fig. 3a, b), differential gene expressions of these motif binding TFs in Naïve, Resolved, and Sensitized differentiated urothelia are visualized as heatmaps. Genes that are not found in RNA-seq data are excluded from the heatmap. (**C)** Top 10 differentially expressed TFs between Sensitized vs. Resolved differentiated urothelia are visualized as a heatmap with Log_2_(FC) and P-adj indicated. Significance of DEGs was determined by Wald test followed by multiple test correction using Benjamini-Hochberg FDR for adjusted p-value.

Despite the large number of epigenetic changes found in sensitized USCs, only a few DEGs were observed when USCs were cultured under stem cell-promoting conditions, suggesting that the epigenetic changes we found may have a greater impact on gene expression upon cell differentiation. Therefore, we performed RNA-seq of differentiated urothelia with or without UPEC infection. PCA of all DEGs showed that the transcriptional profiles of mock-infected differentiated urothelia segregated by infection history (Fig. 4c), indicating intrinsic differences in the differentiated urothelia due to previous infection history. Infection largely caused a uniform shift in the PCA plot for each cell line (Fig. 4c), probably reflecting a conserved transcriptional response to UPEC infection in each convalescent state (Extended Data Fig. 8a). Notably, expression of the Cox-2 encoding gene, *Ptgs2*, was more highly induced upon infection of sensitized and resolved urothelia compared with naïve (Extended Data Fig. 8b), in agreement with a recent in vivo study^7^. Differential gene expression between mock-infected sensitized and resolved differentiated urothelia were visualized in a volcano plot (Fig. 4d), and pathway analysis of DEGs was performed (Fig. 4e). Sensitized differentiated urothelia displayed activation of PTEN signalling, PPARɑ/RXRɑ and Glutathione-mediated detoxification pathways relative to resolved differentiated urothelia, independent of UPEC infection (Fig. 4e and Extended Data Fig. 8c,d). Several of the DEGs were TF genes, including *Klf4* and *Klf5*, (Extended Data Fig. 9), suggesting these may play a role in driving and maintaining unique epigenetic changes in sensitized urothelial cells compared with the other cell types.

### Sensitized USC reprogramming reveals trained immunity signal

Based on our GO pathway analysis data implicating PCD pathways in sensitized-accessible DARs (Fig. 3c) and our in vivo observations that the remodelled sensitized urothelium is characterized by severe exfoliation and Cox-2 inflammation-dependent mucosal wounding during UPEC infection^6^, we specifically interrogated DEGs involved with PCD pathways. A heat map of gene expression shows that many genes associated with PCD pathways were differentially expressed in sensitized and resolved differentiated urothelia relative to each other and to adult naïve differentiated urothelia (Fig. 4f). While *Casp1* (which was the most highly upregulated gene when comparing mock-infected sensitized urothelia to resolved (Supplementary Table 2)) and other pyroptosis-related genes (including *Aim2*, *Gsdmc2* and *Gsdmc3*) were upregulated in sensitized differentiated urothelia, other pyroptosis-related genes (such as the *Naips*) as well as apoptosis and necroptosis-related genes were upregulated in resolved differentiated urothelia, suggesting that resolved and sensitized cells are predisposed to different PCD mechanisms during UPEC infection.

**Fig. 5:**
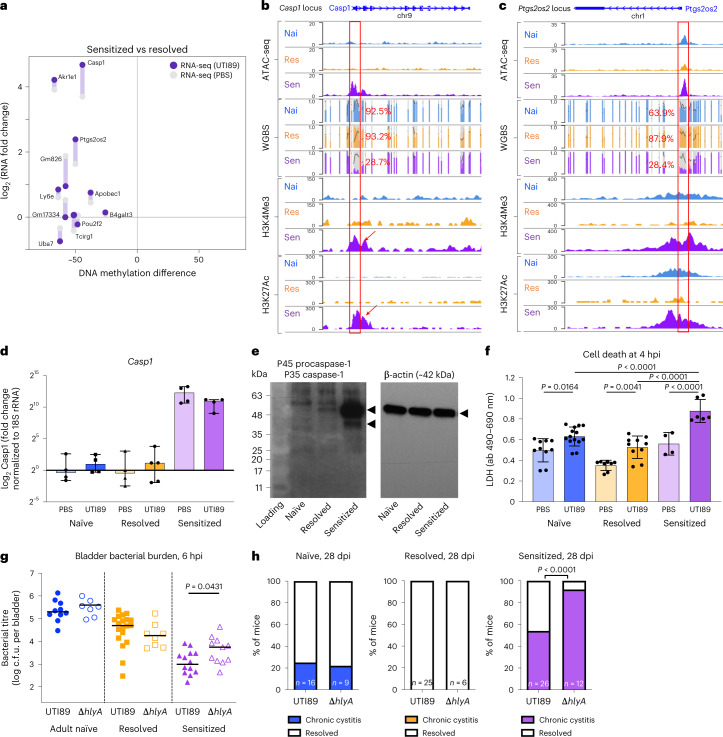
Increased caspase-1-mediated inflammatory cell death in sensitized USCs may protect sensitized mice from acute and chronic UPEC infection. **a**, For those DMRs found within 1 kb of promoter regions, RNA-seq fold changes comparing sensitized vs resolved differentiated urothelia either with (*y* axis, dark purple dots) or without infection (*y* axis, grey dots) were plotted against the DNA methylation differences (*x* axis) between sensitized and resolved USCs. **b**,**c**, Differences in chromatin accessibility (ATAC-seq), DNA methylation (WGBS) and active histone modifications (H3K4me3 and H3K27ac) at the *Casp1* (**b**) and *Ptgs2os2* (**c**) loci in different USC lines were visualized as combined tracks using the WashU Epigenome Browser map. In WGBS data, the average % methylation at the *Casp1* and *Ptgs2os2* promoter sites (red box) are indicated; colour bars represent % methylation, grey backgrounds represent CpGs, and black lines indicate sequencing depth. CpGs within the *Casp1 and* *Ptgs2os2* promoter regions have 8–25x and 18–23x read coverage, respectively. **d**, Gene expression of *Casp1* in differentiated urothelia was measured by RT–qPCR (data from *n* = 4, 4, 4, 5, 4, 4 samples generated from adult naïve, resolved and sensitized differentiated urothelia that are then infected with either PBS or UTI89, respectively, are represented as mean ± s.d.). **e**, Protein expression of caspase-1 using two different cell lines (N2, R2, S2 and N3, R3, S3) was assessed by western blot, and N3, R3 and S3 are represented. **f**, Cell death of differentiated urothelia 4 h after UTI89 infection was measured by LDH assay. Data are mean ± s.d., obtained from *n* = 7, 10, 14, 8, 10, 4, 6 samples from generated from adult naïve, resolved and sensitized differentiated urothelia, 2–3 biologically independent cell lines, per condition, that are then infected with either PBS or UTI89, respectively; significance was determined with a one-way analysis of variance (ANOVA). **g**,**h**, Naïve, resolved and sensitized mice were challenged with 10^7^ c.f.u. of WT UTI89 (HlyA+) or UTI89Δ*hlyA*. Data are combined from 2–3 independent experiments. **g**, Bladder bacterial burdens at 6 hpi (*n* = 10, 7, 19, 8, 14, 11 adult naïve, resolved and sensitized mice challenged with 10^7^ c.f.u. of either UTI89 or UTI89ΔhlyA, respectively) examined over 2–3 independent experiments. Bars indicate median values and two-tailed Mann–Whitney *U* test was used to determine significance. **h**, Incidence of chronic cystitis at 28 dpi. Two-sided Fisher’s exact test; *P* values are indicated when significant. Source data

**Extended Data Fig. 10:**
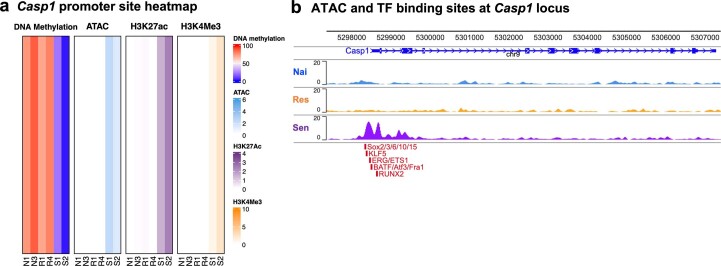
Increased chromatin accessibility for TF binding at *Casp1* locus in Sensitized USCs, related to Figs. 3 and 5. **(A)** Differences in chromatin accessibility, DNA methylation, and histone modifications, H3K4Me3 and H3K27Ac, in different USCs were assessed by ATAC-seq, whole genome bisulfite sequencing (WGBS), and CUT&RUN. Relative DNA methylation, chromatin accessibility, and histone modifications at the *Casp1* promoter site comparing Naïve, Resolved and Sensitized USCs are shown as a heatmap by displaying the normalized signal using read coverage and fraction of reads under consensus peaks. **(B)** Potential TF binding sites near *Casp1* promoter are visualized on the epigenome browser map.

We next examined whether DNA methylation differences in the USCs correlated with RNA-seq fold changes in the differentiated urothelia between sensitized and resolved USCs, focusing on DMRs at promoter sites (Fig. 5a). Most genes, including *Casp1* and *Ptgs2os2* (a positive regulator of Cox-2 expression *in cis* and pro-inflammatory response regulator *in trans*), showed a negative correlation between relative gene expression and the level of DNA methylation at that gene’s promoter site (Fig. 5a). Using the WashU Epigenome Browser, chromatin accessibility, DNA methylation and histone modification marks were visualized at the *Casp1* and *Ptgs2os2* loci (Fig. 5b,c). The *Casp1* and *Ptgs2os2* promoter loci of sensitized USCs are each relatively hypo-methylated while also being enriched in the active histone marks, H3K4Me3 and H3K27Ac, relative to naïve and resolved USCs (Fig. 5b,c and Extended Data Fig. 10a). Several TFs that were enriched in sensitized-accessible DARs (Extended Data Fig. 3a), such as members of the Sox and AP-1 families (Klf5, ETS and RUNX2), have predicted binding sites near the *Casp1* promoter as indicated on the Epigenome Browser map (Extended Data Fig. 10b). *Casp1* expression by RT–qPCR was approximately 1,000-fold higher in sensitized differentiated urothelia compared with resolved or naïve USCs, independent of UPEC infection (Fig. 5d). Concordantly, immunoblot staining showed detectable caspase-1 only in sensitized differentiated urothelia (Fig. 5e), in agreement with our previous ex vivo proteomics of convalescent sensitized mouse urothelium^8^.

In our previous studies, we found that the secreted pore-forming bacterial toxin α-hemolysin (HlyA), commonly produced by UPEC, induces caspase-1 and caspase-11(caspase-4 in humans)-dependent pyroptosis in human and mouse urothelial cells—a protective response that leads to exfoliation of infected cells^22^. We hypothesized that enhanced caspase-1 expression in the sensitized differentiated urothelium would lead to a more robust pyroptotic cell death response upon wild type (WT) (HlyA+) UPEC infection in vitro. A lactate dehydrogenase (LDH) cytotoxicity assay demonstrated that UPEC infection induced cell death in naïve, resolved and sensitized differentiated urothelia (Fig. 5f), but cell death was significantly greater in sensitized differentiated urothelia. In challenge infections using WT UTI89 and UTI89Δ*hlyA* strains in naïve, resolved and sensitized mice, we observed that Δ*hlyA* infection, which does not activate caspase-1-mediated pyroptotic cell death, showed significantly increased bacterial burdens in sensitized mice compared with WT mice, whereas no differences were observed in naïve and resolved mice (Fig. 5g). Furthermore, the incidence of recurrent chronic cystitis at 28 d post-infection was significantly increased in sensitized mice when infected with Δ*hlyA* compared with WT (Fig. 5h), indicating that caspase-1 overexpression in sensitized urothelial cells is a protective response that helps to resolve challenge UPEC infection.

## Discussion

Previous studies have shown that long-lasting bladder tissue remodelling occurs in response to UPEC infection, and this remodelling is accompanied by changes in susceptibility to subsequent infection, depending on previous infection outcomes^5–7^. We hypothesized that this altered susceptibility is mediated, at least in part, by the development of trained immunity at the bladder epithelial mucosa. In contrast with adaptive immunity, which encompasses antigen-specific responses by T and B lymphocytes, ‘trained immunity’ is characterized by antigen non-specific tissue adaptation to acute and chronic inflammation, sometimes in response to infection, and has been predominantly studied in professional innate immune cells such as macrophages, monocytes, dendritic cells and natural killer cells^23–25^. Here we used a primary epithelial cell culture system^10^ to elucidate the urothelial-intrinsic contribution to bladder mucosal remodelling as a consequence of a previous infection, discovering evidence for epigenetic reprogramming of the urothelial stem cells as a mechanism of trained immunity to subsequent urinary tract infection.

The bladder urothelium of previously infected mice is known to be resistant to intracellular colonization relative to age-matched naïve mice^6,7^. However, the mechanism for this intracellular colonization resistance differs between resolved and sensitized mice. In resolved mice, UPEC initially form intracellular bacterial communities in the superficial facet cells, similar to that in adult naïve mice, but they are rapidly shed within the first 6 h of infection via enhanced TNFα-mediated inflammation^7^. In contrast, intracellular bacterial communities do not form at all in sensitized urothelium in vivo, probably due to the small cell size and actin-gating of the incompletely differentiated superficial cells^6,26^. In this work, we have elucidated another facet of bladder colonization resistance in sensitized urothelium, where HlyA-mediated urothelial cell death and exfoliation further reduces early bladder colonization. This effect is probably a consequence of the increased baseline expression of caspase-1 and perhaps other inflammasome-associated factors, such as Gasdermins C2 and C3 that can act as terminal effectors of inflammatory cell lysis^27^, which were observed here in vitro and were previously described in ex vivo proteomics studies of mouse urothelia^8^. *Aim2*, which encodes a cytosolic innate immune sensor that can activate the caspase-1 inflammasome, was also more highly expressed in sensitized differentiated urothelia. In the skin of mice, imiquimod and the resulting inflammation enables the skin to have a more rapid Aim2-mediated response to a secondary inflammatory insult^28^, suggesting that epigenetic reprogramming of inflammasome components may be a common mechanism for priming inflammation sensors to prepare for secondary exposure at barrier tissue sites. Furthermore, in mice, maternal interleukin-6 (IL-6) produced in response to infection can induce epigenetic changes in fetal intestinal epithelial stem cells in utero^29^. In contrast to these previous studies, our work demonstrates a direct role of a mucosal bacterial infection in eliciting specific epigenetic changes to mucosal epithelial stem cells that alter the outcome of subsequent infections.

The protective caspase-1-mediated trained immunity in the sensitized bladder is often overcome by Cox-2-dependent inflammation that occurs in response to high bacterial burdens during the first 24 h of acute rUTI^6,8^. Cox-2 expression occurs mainly at the basal urothelial cell level, but its activity can elicit mucosal wounding through excessive recruitment of neutrophils, thereby transforming the colonization landscape in favour of extracellular colonization and growth of the bacteria. Thus, sensitized mice have competing protective and sensitizing responses to challenge infection, which typically manifest as an extreme bimodal distribution of infection burdens by 24 h post-infection^5^. Although both resolved and sensitized bladders have enhanced early Cox-2 responses in vivo, by 24 h post-infection, the bladder inflammatory response is only sustained in sensitized mice^7^ and Cox-2 inhibition protects sensitized mice against severe recurrent cystitis^6,8^. Similarly, infection-induced *Ptgs2* gene (encoding Cox-2) expression was enhanced in both sensitized and resolved differentiated urothelia relative to naïve. However, the expression of the *Ptgs2os2* gene, which encodes LincRNA-Cox-2, a positive regulator of *Ptgs2* expression and general inflammation^30^, differed between these cell lines, being increased in sensitized relative to resolved differentiated urothelia. Concordantly, we found differential epigenomic marks that were associated with increased accessibility of the *Ptgs2os2* locus in the sensitized USCs, suggesting that *Ptgs2os2* expression in differentiated urothelia is altered by epigenetic changes in the USCs. Thus, epigenetic changes to the *Ptgs2os2* locus may play a role in promoting the sustained pro-inflammatory responses of the sensitized bladder, thus overcoming the protective function of increased *Casp1* expression.

Changes in expression of DNA methyltransferases, which methylate CpG sites of DNA, have been implicated as a mechanism for remodelling the epigenome in response to acute UPEC infection^31^, potentially explaining how a previous infection, whether self-resolving or chronic, could alter the USC epigenome. However, chronic inflammation itself is also a potent inducer of epigenetic memory that could explain the differences in epigenetic marking between resolved and sensitized USCs. One model for this rewriting of the epigenome is the presence of so-called ‘memory domains’ where ‘pioneer’ TF binding to nucleosomes in response to stimuli opens up chromatin in stress-responsive loci and allows epigenetic writers to remodel the chromatin to allow it to remain open after the stimuli are removed^32^. Klf4, which was upregulated in sensitized differentiated urothelia compared with resolved urothelia, is a known pioneer TF^33^. Further, our motif discovery analyses support the hypothesis that epigenetic remodelling in the USCs occurs primarily at AP-1-associated DARs in response to a self-limiting acute infection (resolved-accessible DARs), but that severe acute infection leading to chronic infection and inflammation (sensitized-accessible DARs) induces epigenetic remodelling not only at AP-1-associated DARs, but also at additional TF-associated DARs, such as those with Klf and Sox family motif sites.

Our discovery of epithelial stem cell epigenetic reprogramming upon UPEC infection has implications for understanding the mechanism of epithelial-intrinsic trained immunity against not only UTIs, but also other types of infection or inflammatory disease. Further mechanistic studies may lead to novel therapies for a range of recurrent infections and inflammatory diseases. For example, therapeutic use of an inhibitor of histone demethylase LSD1, which is overexpressed in skin epithelial cancer, drives notable increases in H3K4 methylation in the cells, thus leading to both premature epidermal differentiation and the repression of squamous cell carcinoma^34^. Therefore, further investigation to identify which epigenetic factors, TFs or inflammatory mediators are directly responsible for establishing and maintaining these specific epigenetic memories in vivo would provide deeper mechanistic insights and shed light on potential therapeutic targets to prevent rUTIs and/or reverse the epigenetic imprinting that leads to increased susceptibility to recurrent disease.

## Methods

### Ethics statement

All animal experimentation was conducted according to the National Institutes of Health guidelines for the housing and care of laboratory animals. All experiments were performed in accordance with institutional regulations after review and approval by the Animal Studies Committee at Washington University School of Medicine in St Louis, Missouri.

### Bacterial strains

The UPEC strains used in this study were the human cystitis isolate UTI89 and derivative thereof: UTI89 attHK022::COM-GFP (UTI89-KanR)^35^, UTI89 pANT4 and UTI89 hlyA::KD13 (UTI89 Δ*hlyA*-KanR)^22^. For both mouse and in vitro infection, UTI89 strains were cultured statically in lysogeny broth (LB) at 37 °C overnight, subcultured 1:1,000 into fresh media and cultured statically at 37 °C for 18 h.

### Mouse infections

Female C3H/HeN mice (Envigo) were 7–8 weeks old (‘juvenile’) at the time of the initial infection. A total of 10^8^ c.f.u. of UTI89 were inoculated into the bladder of C3H/HeN mice by transurethral catheterization^5,36^. C3H/HeN mice develop chronic cystitis in an infection dose-dependent manner and this inoculum results in chronic cystitis in ~50% of mice^6^. To monitor infection outcomes, urine was collected. Persistent bacteriuria (10^4^ c.f.u. ml^−1^) is defined as a specific and sensitive cut-off for detecting chronic cystitis^5^. Chronic cystitis during initial infection was defined as persistent high bacteriuria (>10^4^ c.f.u. ml^−1^ urine) at every timepoint urine was collected (1, 3, 7, 10, 14, 21 and 28 d post-infection), while resolution of cystitis was defined as urine bacterial titre dropping below this cut-off in at least one timepoint.

At 4 weeks post-infection, all mice were treated with trimethoprim and sulfamethoxazole in the drinking water for 10 d (54 and 270 μg ml^−1^ water, respectively)^6^. Urine was collected weekly to confirm clearance of bacteriuria. Four weeks after the initiation of antibiotics, naïve, resolved and sensitized mice were used to isolate primary USCs or used for secondary infection assay. For the secondary infection, mice were challenged with 10^7^ c.f.u. of bacteria inoculated into the bladders, then humanely euthanized at 6 h post-infection, and bacterial burdens were determined to assess acute outcomes.

### Cell line culture

Human bladder carcinoma epithelial cells, designated 5637 (ATCC HTB-9) cells, were cultured in RPMI-1640 medium containing 10% FBS at 37 °C in the presence of 5% CO_2_.

### Primary USC isolation and culture

Bladder tissue from juvenile, convalescent naïve, resolved and sensitized mice were isolated, bisected and incubated in stripping solution at 4 °C overnight. The urothelial cells were scraped off from the bladder tissue, spun down at 4 °C at 300 *g* for 5 min, resuspended in fresh collagenase IV solution and incubated with rocking at 37 °C for 20 min. The cells were disaggregated by gentle pipetting, filtered with a 100 μm strainer, then washed with washing media. The cells were cultured in matrigel (BD Biosciences) with 50% L-WRN CM containing 10 mM Y-27632 and 10 mM SB431542 (R&D System)^9^. Media were changed every 2 d and cells were passaged every 3 d (1:2–3 split). USCs were used for experiments after 10 passages to remove any remaining non-stem urothelial cells.

### Differentiated urothelium culture on transwell

USCs were washed in PBS with 0.5 mM EDTA, trypsinized in 0.05% Trypsin and 0.5 mM EDTA for 1 min at 37 °C, dissociated by vigorous pipetting, filtered through a 40 μm cell strainer and resuspended in washing media. Transwells (Corning Costar, 3413) were coated in PBS with 1:40 Matrigel for 30 min at 37 °C. Then 3–4 × 10^4^ USCs were seeded on the transwell insert, and 100 μl and 600 μl 50% CM containing 10 mM Y-27632 were added to the apical and basolateral compartments of the transwell, respectively.

### TER measurements

Resistance of the urothelial multilayers was assessed by TER measurement using an epithelial volt-ohm metre (World Precision Instruments). The average value of triplicate measurements was multiplied by the area of the transwell membrane (0.33 cm^2^) to obtain a final value in ohm × cm^2^ (ref. ^37^).

### In vitro UPEC infection assay

When urothelium was fully differentiated (TER value >4,000 ohm × cm^2^), cultures were washed 3 times in warm DMEM/F12 media and infected with UPEC strains at multiplicity of infection 10. Transwells were then incubated at 37 °C for 30 min, changed to media containing 100 μg ml^−1^ gentamicin to clear the extracellular bacteria and cultured for an extended time. After infection, apical and basolateral media were spun down at 2,000 *g* at 4 °C for 5 min and used for LDH assay (TaKaRa, MK401). Transwells were washed with sterile PBS, then used for various analyses.

### Whole-mount confocal staining

Differentiated urothelia on transwells were washed and fixed in PBS with 4% paraformaldehyde for 15 min and rinsed 3 times with PBS. Subsequently, 100 μl 0.2% Triton X was added for 10 min then dumped and the cells were incubated in 100 μl 2% BSA for blocking for 30 min. The samples were stained with primary antibody, mouse monoclonal anti-keratin 20 (Abcam, ab854, 1:200) and secondary antibody, Alexa Fluor 647 donkey anti-mouse IgG (Invitrogen, A-31571, 1:1,000), then further stained with Alexa Fluor 555 Phalloidin (ThermoFisher, A34055, 1:200) and 4′,6-diamidino-2-phenylindole (DAPI) (ThermoFisher, D1306, 1:1,000). For confocal microscopy,va ZEISS LSM880 laser scanning microscope with Airyscan was used. Fiji ImageJ and macro programme were used to automatically calculate urothelial cell surface area in *z*-stacked confocal images.

### Histopathology and immunofluorescence

USCs or differentiated urothelia were fixed overnight in 10% formaldehyde at 4 °C. After washing in PBS, the fixed samples were pre-embedded into 2% agar, cut vertically, put in transwells side face up, embedded again in paraffin blocks and sectioned. The slides were stained for H&E and immunostained for selected antibodies. For immunofluorescence staining, slides were deparaffinized, hydrated, blocked with 10% heat-inactivated horse serum (HIHS) and 0.3% Triton X-100 in PBS, incubated with primary antibody in 1% HIHS and PBS overnight at 4 °C and secondary antibody in PBS for 30–60 min at room temperature^6^. The primary antibodies used were mouse monoclonal anti-keratin 20 (Abcam, ab854, 1:200), goat polyclonal anti-E-cadherin (R&D Systems, AF748, 1:500), goat polyclonal anti-uroplakin 3a (Santa Cruz, sc-15186, 1:500), mouse monoclonal anti-uroplakin 3a (Fitzgerald, 10R-U103a, 1:50), rabbit polyclonal anti-p63 (GeneTex, GTX102425, 1:1,000), rabbit monoclonal anti-K5 (Abcam, ab150074, 1:100) and mouse monoclonal anti-keratin 14 (Santa Cruz, sc-53253, 1:50). Alexa Fluor secondary antibodies and DAPI were used at 1:1,000 dilution. Further antibody information is provided in Supplementary Table 3. Samples were visualized on a Zeiss Axio Imager M2 Plus wide-field fluorescence microscope.

### Scanning electron microscopy (SEM)

Differentiated urothelia were washed 3 times in PBS, fixed in EM fixative (2% paraformaldehyde, 2.5% glutaraldehyde in 1× PBS) for 1 h on ice and washed 3 times in PBS. Samples were then post-fixed in 1.0% osmium tetroxide, dehydrated in increasing concentrations of ethanol, then dehydrated at 31.1 °C and 1,072 p.s.i. for 16 min in a critical point dryer^6^. Samples were mounted on carbon tape-coated stubs and sputter-coated with gold/palladium under argon^6^, then imaged on a Zeiss Crossbeam 540 FIB-SEM.

### RNA isolation and RT–qPCR

RNAs were extracted from USCs or differentiated urothelia using RNAeasy Plus mini kit (Qiagen) and reverse-transcribed with iScript Reverse Transcription Supermix (BioRad). We used 1 μl 12.5 ng μl^−1^ ccomplementary DNA with intron-spanning primers specific to each gene, and iQ SYBR Green Supermix was used according to the manufacturer’s instructions (BioRad). Sequences of the primers we used in this study are listed in Supplementary Table 4. Expression values were normalized to 18S, and relative expression compared to control was determined by the cycle threshold (ΔΔCt) method^38^. Each sample was run in triplicate, and average Ct values were calculated.

### RNA-seq and data analysis

Illumina cDNA libraries were generated using a modified version of the RNAtag-seq protocol^39^. Briefly, 1 μg of total RNA was fragmented, depleted of genomic DNA, dephosphorylated and ligated to DNA adaptors carrying 5’-AN_8_-3’ barcodes of known sequence with a 5’ phosphate and a 3’ blocking group. Barcoded RNAs were pooled and depleted of ribosomal RNA using the Ribo-Zero rRNA depletion kit (Illumina). cDNA libraries were generated by adding a second adaptor by template switching and PCR amplification with primers carrying Illumina P5 or P7 sequences, then the libraries were sequenced on the Illumina HiSeq 2500. Paired-end sequencing reads in a pool were demultiplexed on the basis of their associated barcode sequence using custom scripts (https://github.com/broadinstitute/split_merge_pl). Reads were then trimmed using cutadapt v1.6 and trimmed reads were aligned to the *Mus musculus* mm10 genome using tophat2 v2.0.11 and bowtie2 v2.2.2. Gene counts were conducted by HTSeq v0.6.0 and read counts were assigned to annotated transcripts using Salmon v0.8.2^7^.

Read normalization and differential expression were conducted with DESeq2 v1.14.0^40^. rlog transformations of DESeq-normalized reads were used for PCA plots. Fragments per kilobase of transcript per million mapped reads (FPKM) normalization of DEseq2 reads was used for *z*-score heat maps. TF expression was determined using DESeq2 FPKM-normalized values and a list of mouse TFs (*n* = 453) from HOCOMOCO v11^41^, a TF database of validated TF motifs. An adjusted *P* value cut-off of 0.05 was used and TF candidate expression was visualized using *z*-score heat maps. Statistically significant differences in gene expression were assessed by the Wald test, followed by multiple test correction using Benjamini-Hochberg false discovery rate (FDR), with adjusted *P* < 0.05 being considered significant. Pathway analyses were performed with ingenuity pathway analysis (IPA). Significance was determined by a right-tailed Fisher’s exact test, with *P*_adj_ < 0.05 being considered significantly enriched pathways.

### ATAC-seq and data analysis

Single cells (1–2 × 10^5^) of naïve, resolved and sensitized USCs were used for nuclei preparation, and 50,000 nuclei were counted and transferred into 25 μl of 2× TD buffer. Omni-ATAC-seq reaction mix (25 μl) including TDE1 enzyme was added to 25 μl of 50,000 nuclei in 2× TD buffer, then the samples were incubated at 37 °C for 30 min (tapped every 10 min during the incubation in a heat block). Transposed DNA fragments were immediately purified using a MinElute PCR purification kit (Qiagen). ATAC-seq libraries were amplified by PCR amplification (10–12 cycles) with an initial 5 min extension at 72 °C and purified using AMPure XP beads (Beckman Coulter). The purified libraries were eluted with 20 μl of nuclease-free water, quantified using Qubit dsDNA HS assay kit (ThermoFisher), and their size distribution checked with a 4200 TapeStation (High Sensitivity D1000 ScreenTape and Reagents). Paired-end ATAC-seq libraries were sequenced on an Illumina NextSeq 500 (~350 million reads).

ATAC-seq analysis^42^ used the following tools and versions: Fastqc v0.11.5, Cutadapt v1.11, Samtools v1.5, Bowtie2 v2.3.0, picard v2.10.0, Macs2 v2.1.1.20160309 and bedtools v2.26.0. Sequencing reads were demultiplexed using sample-specific index sequences, quality checked with fastqc, trimmed using cutadapt and aligned to a reference mouse genome (mm10) using bowtie2^43^. Picard was then used to remove secondary alignment, multiply mapped reads and PCR duplicated reads, and peak calling was done with MACS2^44^. Irreproducible discovery rate (IDR) analysis with two replicates was performed following ENCODE’s guidelines^45^, and ATAC peaks with IDR < 0.05 were chosen as highly reproducible accessible chromatin regions for further analysis. The ATAC-seq signals were visualized on the WashU Epigenome Browser^46^ as fold change (FC) over background using bedGraph tracks generated using the MACS2 bdgcmp function.

To identify DARs, Diffbind v2.10.0 was used on IDR < 0.05 ATAC peaks, and Benjamini-Hochberg FDR with a cut-off <0.05 was used for statistical significance. Significant DARs (FDR < 0.05) were used for generating volcano plots and heat maps. GREAT^16^ (basal plus extension parameter) was used for GO pathway analysis. GREAT ranks results by binomial *P* value using a binomial test. Sensitized (FC > 1.5) and resolved-specific DARs (FC < −1.5) were separately analysed and the top 15 enriched pathways are shown in Fig. 3e–f.

### WGBS and data analysis

Single cells (1–2 × 10^5^) of USCs were treated with DNase I to remove trace DNA contamination from the Matrigel. Genomic DNA (gDNA) was prepared from the cells using DNeasy Blood & Tissue kit (Qiagen, 69504). Using 200 ng of gDNA and 0.4 ng lambda, DNA was bisulfite treated using EZ DNA Methylation-Direct kit (Zymo, D5020) and processed with xGen Methyl-Seq Library Prep kit (IDT, 10009824) to generate Illumina-compatible WGBS libraries. The libraries were sequenced on a NovaSeq S4 300XP (~300 million reads) by MGI institute.

WGBS analysis commands with specific parameters are detailed in the Code availability section. Briefly, fastqQC v0.11.8 was used to assess the quality of the raw reads. Subsequently, the paired-end reads were trimmed to remove adaptor sequences and low-quality reads with Cutadapt v1.18 and reassessed using FastqQC. The mouse reference genome mm10 was first bisulfite converted using Bismark v0.20.0. The paired-end reads were aligned to the mm10 bisulfite-converted genome and deduplicated using ‘deduplicate_bismark’. DNA methylation levels were calculated using ‘bismark_methylation_extractor’ and displayed in a methylC format on the WashU Epigenome Browser^46^. Bisulfite conversion was estimated using the conversion rate of cytosine to thymine in the lambda reference genome.

DMRs were identified with DSS v2.43.2^47^ using a two-group comparison for biological replicates and called using ‘DMLtest’ and ‘callDMR’. A PCA plot of CpG methylation within DMRs was generated using Deeptools v3.3.0. Biological replicates were combined by merging fastq files between replicates and reprocessing using the steps previously described. CpG density was visualized using a 5x coverage cut-off and ggplot2 v3.3.6.

Sensitized-specific DMRs were defined as the overlapping regions between naïve vs sensitized and resolved vs sensitized DMRs. The percent methylation for sensitized-specific DMRs was visualized using the R package ‘ComplexHeatmap’. The DNA methylation over sensitized-specific hypo-DMRs were plotted using Deeptools and visualized using ggplot2. Overlapping regions between DMRs were identified and visualized using Intervene^48^ ‘Venn’ with default parameters. GREAT^16^ analysis on sensitized hypo-DMRs was performed as described in ATAC analysis. The sensitized hypo-DMRs were also analysed for genomic annotation using UCSC (https://genome.ucsc.edu/cgi-bin/hgTables) to download GENCODE M25 (https://www.gencodegenes.org/mouse/release_M25.html). The promoter was defined as 1 kb upstream of transcription start site. Genomic annotation priority was assigned in the following order: promoter, coding exon, 5’ UTR, 3’ UTR, intron and intergenic. DMRs were assigned to annotation if the DMR overlapped 20% of the annotation using BEDTools v2.27.1 intersect and were plotted using DNA methylation percent change between sensitized and resolved against the log_2_(FC) of associated genes between sensitized and resolved differentiated urothelia with or without infection.

### CUT&RUN and data analysis

Single cells (0.2 × 10^6^) of USCs were slightly crosslinked in 0.1% formaldehyde and fixed cell pellets were stored at −80 °C before use. H3K4Me3, H3K27Ac and H3K27Me3 CUT&RUN was performed using CUT&RUN assay kit (Cell Signaling, 86652), with a few modifications. Briefly, cells were attached to concanavalin A beads for each experiment. Cells were permeabilized with digitonin in the antibody binding buffer containing spermidine and protease inhibitors and then incubated with primary antibodies against H3K4Me3 (Cell Signaling, 9751, 1:50), H3K27Ac (Cell Signaling, 8173, 1:100) or H3K27Me3 (Cell Signaling, 9733, 1:50) at 4 °C overnight on a rotator. The beads–cells mixture was washed 3 times with digitonin buffer, resuspended in 50 µl pAG-MNase and incubated at 4 °C for 1 h on a rotator. Samples were digested in PCR tubes containing 150 µl cold digitonin buffer and 3 µl CaCl_2_ at 4 °C for 30 min in a thermal cycler. Then, beads were transferred back to the microcentrifuge tubes, 150 µl of 1× STOP buffer was added and tubes were incubated at 37 °C for 10 min. Placing tubes on a magnetic rack, supernatants were collected to a new tube. Crosslinks were reversed by adding 3 µl 10% SDS solution and 2 µl 20 mg ml^−1^ proteinase K, then samples were incubated at 65 °C for 2 h. DNA from enriched chromatin samples were purified using DNA spin columns (Zymo, D4013). The sequencing library was prepared with Ultra II DNA Library Prep kit (NEB, E7645) following the manufacturer’s instructions, but reducing the anneal and extension time to 10 s during PCR enrichment of adaptor-ligated DNA.

A detailed list of commands and parameters can be found under Code availability. Briefly, fastqQC v0.11.9 was used to assess the read quality. Subsequently, the paired-end reads were trimmed with Cutadapt v1.9 and reassessed using fastqQC. Reads were then aligned using bowtie2 v2.3.4.1^49^. Mitochondrial reads were removed using samtools v1.9 and deduplicated using Picard v2.8.1 MarkDuplicates. Uniquely mapped reads were extracted using samtools view. Peaks were called using MACS2 v2.1.1.20160309 ‘callpeak’: ‘-q 0.01’ for narrow peaks H3K4Me3 and H3K27Ac, and ‘-q 0.05 --broad’ for H3K27Me3. Encode-defined blacklisted regions were removed. For each histone modification, a consensus peak list was used to calculate the fraction of reads in peaks (FRIP). Reads then were converted to bigWig format using Deeptools and normalized using read coverage and FRIP score. The normalized biological replicates were combined using ucsc-bigwigmerge v377 and converted from bedGraph to bigWigs using kentUCS v334 and mm10 chromosome sizes from UCSC (http://hgdownload.cse.ucsc.edu/goldenPath/mm10/bigZips/mm10.chrom.sizes). To profile sensitized-specific DMR regions for other epigenetic modifications, the sensitized hypo-DMRs were overlapped with MASC2 narrow peaks for ATAC, H3K4Me3 and H3K27Ac, and broad peaks for H3K27Me3. The corresponding peak score was normalized using the FRIP scores and plotted using the R package ‘ComplexHeatmap’. Using the normalized bigWig tracks, ATAC, H3K4Me3, H3K27Ac and H3K27Me3 signals were plotted over the sensitized-specific hypo-DMRs using Deeptools. The normalized bigWig signals were used for the *casp1* heat map.

### Immunoblotting

Cells were lysed with cell lysis buffer (Cell Signaling, 9803S) according to the manufacturer’s instructions. Rapid Gold BCA Protein Assay kit was used to determine protein concentrations in the cell lysate, and equal amounts of protein were separated by SDS–PAGE and transferred to a nitrocellulose membrane. Membranes were incubated overnight with primary antibodies against caspase-1 (AdipoGen, AG-20B-0042-C100, 1:5,000) and β-actin (MA5-15739, Invitrogen, 1:10,000). HRP-linked secondary antibody (Cell Signaling, 7076S, 1:3,000) and ECL reagent (Amersham, RPN2209) were used to visualize protein bands.

### Statistics and reproducibility

For representing images of confocal, immunofluorescence staining and SEM, 3–4 different cell lines (among J1-5, N1-4, R1-4 and S1-4) were stained and imaged. For western blot images, two different cell lines were tested. Statistics for plots/graphs were analysed in GraphPad Prism v8.4.3. Exact *P* values are indicated when significant (*P* < 0.05) (GraphPad Prism did not provide exact *P* value when *P* < 0.0001).

### Reporting summary

Further information on research design is available in the Nature Portfolio Reporting Summary linked to this article.

## Supplementary information

Reporting Summary

Supplementary Tables1–4 Table 1. List of 2,880 significantly differentially accessible regions (DARs) between sensitized and resolved USCs and their proximal gene names, supporting Fig. 3a,b. Table 2. Top differentially expressed genes (DEGs) from IPA comparing mock-infected sensitized vs resolved differentiated urothelia. ‘Exp log ratio’ indicates expression log_2_ fold change. Table 3. Antibody information. Table 4. List of qRT–PCR primers.

## Data Availability

The data supporting the findings of this study are available within the paper, its Supplementary Information, or Source Data. RNA-seq, ATAC-seq, WGBS and CUT&RUN data have been deposited at NCBI under BioProject ID no. PRJNA705407. WashU Epigenome Browser map visualizing ATAC-seq, WGBS-seq and CUT&RUN, and RNA-seq (forward-strand: green and reverse-strand: orange) data are accessible at the following links: Combined replicates: https://epigenomegateway.wustl.edu/browser/?genome=mm10&noDefaultTracks=1&hub=https://wangftp.wustl.edu/~jharrison/PUBLISHED_DATAHUBS/Hultgren/Russell_Bacterial_infection_combined.json Combined and individual replicates: https://epigenomegateway.wustl.edu/browser/?genome=mm10&noDefaultTracks=1&hub=https://wangftp.wustl.edu/~jharrison/PUBLISHED_DATAHUBS/Hultgren/Russell_Bacterial_infection_all.jsonSource data are provided with this paper.

## Source data

Source Data Fig. 1 Tab 1 (Fig. 1b). Measurement of TER at indicated days during 21 d of culture/differentiation of #1–5 independent juvenile C3H/HeN in transwells.
Source Data Fig. 2 Tab 1 (Fig. 2b). Urine bacterial titre time course over 4 wpi during initial infection with 108 c.f.u. UTI89 KanR in 8 weeks of female C3H/HeN mice. Tab 2 (Fig. 2f). Average cell size per transwell represented as median with 95% CI. Tab 3 (Fig. 3g). Individual cell size represented as median with 95% CI.
Source Data Fig. 3 Tab 1(Fig. 3c). Sensitized-accessible DARs (sensitized vs resolved comparison, *n* = 747 DARs, fold change >1.5, FDR < 0.05) used for GREAT GO term analysis. Tab 2 (Fig. 3d–h). List of 189 sensitized-specific differentially methylated regions (DMRs) from naïve vs sensitized and resolved vs sensitized comparisons.
Source Data Fig. 4 Tab 1 (Fig. 4b-1). DEG comparison between sensitized vs resolved USCs, also supporting ED Fig. 6b. Tab 2 (Fig. 4b-2). DEG comparison between sensitized vs naïve USCs, also supporting ED Fig. 6a. Tab 3 (Fig. 4d). DEG comparison between mock-infected sensitized vs resolved differentiated urothelia used for volcano plot. Tab 4 (Fig. 4e). IPA pathway analysis of mock-infected sensitized vs resolved differentiated urothelia. Tab 5 (Fig. 4f). A heat map visualization of differential gene expression in naïve, resolved and sensitized differentiated urothelia with or without infection, also supporting Extended Data Figs. 8b and 9.
Source Data Fig. 5 Tab 1 (Fig. 5d). Gene expression of *Casp1* in naïve, resolved and sensitized differentiated urothelia with or without UTI89 infection was measured by qRT–PCR (*n* = 4, 4, 4, 5, 4, 4). Tab 2 (Fig. 5f). LDH measurement of naïve, resolved and sensitized differentiated urothelia upon UTI89 infection at 4 hpi. Data obtained from *n* = 7, 10, 14, 8, 10, 4, 6. Tab 3 (Fig. 5g). Naïve, resolved and sensitized mice were challenged with 10^7^ c f u of WT UTI89 (HlyA+) or UTI89Δ*hlyA*. Data were combined from two to three independent experiments. Bladder bacterial burdens at 6 hpi, measured from *n* = 10, 7, 19, 8, 14, 11 mice, respectively, were examined over 2–3 independent experiments. Tab 4 (Fig. 5h). Incidence of chronic cystitis vs resolution at 28 dpi for naïve, resolved and sensitized mice upon challenge infection.
Source Data Fig. 5 Fig. 5e. Uncropped gel and blot images of N3, R3 and S3 differentiated urothelia. **a**, Whole gel loaded with two sets of 6 samples (PBS mock-infected N3, R3 and S3 urothelial cells and UTI89-infected N3, R3 and S3 urothelial cells) with Tris-glycine 4–20% loading dye. Both uninfected and infected sensitized urothelial cells had caspase-1 staining. Only mock-infected N3, R3 and S3 data were used for the paper since sensitized cells expressed caspase-1 regardless of infection condition. **b**, Membrane image (1–6 lane cut was used for β-actin staining and 7–14 lane cut was used for caspase-1 staining). **c**, Film development was done separately since β-actin and caspase-1 staining needed different exposure times (1 min and 3 min, respectively). β-actin (~48 kDa) expression was similar among all the samples, while only sensitized cells had staining at P45 pro-caspase-1 (~45 kDa) and P35 caspase-1 (~35 kDa) locations. For Fig. 5e in the manuscript, (**d**) 20 s exposure image was used instead of 1 min exposure for β-actin for cleaner band separation.
Source Data Extended Data Fig. 1 Tab 1 (ED Fig. 1b). *p63* expression measured by qRT–PCR from C3H/HeN primary cells cultured in the indicated condition in ED Fig. 1b–d. Each condition at measurement time points is *n* = 12, 9, 13, 12, 12, 11, 11, respectively. Tab 2 (ED Fig. 1c). *Axin2* expression measurement using the same set of samples. Tab 3 (ED Fig. 1d). *Upk3a* expression measurement using the same set of samples.
Source Data Extended Data Fig. 2 Tab 1 (ED Fig. 2a). Transepithelial electrical resistance (TER) comparison between primary C3H/HeN USCs and 5637 cells for 2–3 weeks culture in differentiation condition. Tab 2 (ED Fig. 2b). Surface cell size comparison between primary C3H/HeN urothelial cells and 5637 cells (*n* = 10 each).
Source Data Extended Data Fig. 4 Tab 1 (ED Fig. 4a-1). Naïve vs sensitized DMRs. Tab 2 (ED Fig. 4a-2). Naïve vs Resolved DMRs. Tab 3 (ED Fig. 4a-3). Resolved vs sensitized DMRs.
Source Data Extended Data Fig. 6 Tab 1 (ED Fig. 6c). IPA pathway analysis of sensitized vs naïve USCs. Tab 2 (ED Fig. 6d). IPA pathway analysis of sensitized vs resolved USCs.
Source Data Extended Data Fig. 7 Tab 1 (ED Fig. 7a). DEGs comparing juvenile naïve vs adult naïve USCs used for volcano plot.
Source Data Extended Data Fig. 8 Tab 1 (ED Fig. 8a-1). DEG comparison between UTI89-infected vs mock-infected naïve differentiated urothelia, used for volcano plot. Tab 2 (ED Fig. 8a-2). DEG comparison between UTI89-infected vs mock-infected resolved differentiated urothelia, used for volcano plot. Tab 3 (ED Fig. 8a-3). DEG comparison between UTI89-infected vs mock-infected sensitized differentiated urothelia, used for volcano plot. Tab 4 (ED Fig. 8b). DEG comparison between UTI89-infected sensitized vs resolved differentiated urothelia, used for volcano plot. Tab 5 (ED Fig. 8c). IPA pathway analysis of UTI89-infected sensitized vs resolved differentiated urothelia.

## Code availability

ATAC-seq general pipeline:

https://www.encodeproject.org/documents/c008d7bd-5d60-4a23-a833-67c5dfab006a/@@download/attachment/ATACSeqPipeline.pdf

CUT&RUN general pipeline:

https://github.com/Yonghao-Holden/tricks/blob/main/cuttag_pipe_v1.sh

WGBS general pipeline:

https://github.com/hyungjoo-lee/wgbs

The custom code for ATAC-seq, GUT&RUN, WGBS and figures:

https://github.com/jharrison0123/Uropathogenic-Escherichia-coli-infection-induced-trained-immunity-affects-urinary-tract-disease

The code for the volcano plots: https://github.com/bsolson/Volcano_plot

EnhancedVolcano code: https://github.com/kevinblighe/EnhancedVolcano
