# Supplementary material for: Uropathogenic Escherichia coli infection-induced epithelial trained immunity impacts urinary tract disease outcome
Source: Nat Microbiol. 2023 Apr 10;8(5):875–88. doi: 10.1038/s41564-023-01346-6 (PMC10159856; doi:10.1038/s41564-023-01346-6)
Supplement: Supplementary file 1 — Reporting Summary [file 41564_2023_1346_MOESM1_ESM.pdf]

nature portfolio

Corresponding author(s): Scott J. Hultgren, Thomas J. Hannan, Ting Wang

Last updated by author(s): 02.09.2023

Reporting Summary

Nature Portfolio wishes to improve the reproducibility of the work that we publish. This form provides structure for consistency and transparency in reporting. For further information on Nature Portfolio policies, see our Editorial Policies and the Editorial Policy Checklist.

Statistics

For all statistical analyses, confirm that the following items are present in the figure legend, table legend, main text, or Methods section.

n/a	Confirmed	
☐	☒	The exact sample size (*n*) for each experimental group/condition, given as a discrete number and unit of measurement
☐	☒	A statement on whether measurements were taken from distinct samples or whether the same sample was measured repeatedly
☐	☒	The statistical test(s) used AND whether they are one- or two-sided<br>*Only common tests should be described solely by name; describe more complex techniques in the Methods section.*
☐	☒	A description of all covariates tested
☐	☒	A description of any assumptions or corrections, such as tests of normality and adjustment for multiple comparisons
☐	☒	A full description of the statistical parameters including central tendency (e.g. means) or other basic estimates (e.g. regression coefficient) AND variation (e.g. standard deviation) or associated estimates of uncertainty (e.g. confidence intervals)
☐	☒	For null hypothesis testing, the test statistic (e.g. *F*, *t*, *r*) with confidence intervals, effect sizes, degrees of freedom and *P* value noted<br>*Give P values as exact values whenever suitable.*
☒	☐	For Bayesian analysis, information on the choice of priors and Markov chain Monte Carlo settings
☒	☐	For hierarchical and complex designs, identification of the appropriate level for tests and full reporting of outcomes
☐	☒	Estimates of effect sizes (e.g. Cohen's *d*, Pearson's *r*), indicating how they were calculated

Our web collection on statistics for biologists contains articles on many of the points above.

Software and code

Policy information about availability of computer code

Data collection	No software was used for data collection.
Data analysis	RNA-seq reads from each sample in a pool were demultiplexed based on their associated barcode sequence using custom scripts (https://github.com/broadinstitute/split_merge_pl). Barcode sequences were removed from the first read as were terminal G's from the second read that may have been added during template switching. Reads were then trimmed by cutadapt twice (cutadapt-v1.6), once by base quality and once by polyA or polyT repeats. Trimmed reads were then aligned to the Mus musculus mm10 genome using tophat2 (tophat2-v2.0.11, bowtie2-2.2.2). Gene counts were conducted by HTSeq (HTSeq-v0.6.0, options: –format = bam –order = name –stranded = no –idattr = gene_id –mode = union) and read counts were assigned to annotated transcripts using Salmon_0.8.2. Read normalization and differential expression were conducted with DESeq2_1.14.0. rlog transformations of DESeq normalized reads were used for PCA plots. FPKM normalization of DEseq2 reads was used for z-score heatmaps. TF expression was determined using DESeq2 FPKM normalized values and list of mouse TFs (n=453) from HOCOMOCO v11, a TF database of validated TF motifs. An adjusted p-value cutoff of 0.05 was used and TF candidate expression was visualized using z-score heatmaps. Pathway analyses were performed with Ingenuity Pathway Analysis (IPA).<br><br>ATAC-seq analysis used the following tools and versions: Fastqc v0.11.5, Cutadapt v.1.11, Samtools v1.5, Bowtie2 v2.3.0, picard v2.10.0, Macs2 v2.1.1.20160309, bedtools v2.26.0. Sequencing reads were de-multiplexed by using sample-specific index sequences, quality checked with fastqc, trimmed by using cutadapt, and aligned to a reference mouse genome (mm10) by using bowtie2 with these parameters: --local -X 2000 --mm. Picard was then used to remove secondary alignment, multiply mapped reads, and PCR duplicated reads, and peak calling was done with MACS2, with these parameters: -g 1.87e9 --keep-dup all -B --SPMR --nomodel --extsize 73 --shift -37 -p 0.01 --call-summits. Irreproducible discovery rate (IDR) analysis with two replicates was performed following ENCODE's guidelines (link: https://www.encodeproject.org/documents/c008d7bd-5d60-4a23-a833-67c5dfab006a/@@download/attachment/ATACSeqPipeline.pdf), and ATAC peaks with IDR < 0.05 were chosen as highly reproducible accessible chromatin regions for further analysis. The ATAC-seq signals were visualized on the WashU Epigenome Browser as fold change over background using bedGraph tracks generated using the MACS2 bdgcmp

function with this parameter: -m FE.
To identify differentially accessible regions (DARs), Diffbind v2.10.0 was used on the set of ATAC peaks (IDR < 0.05) with these parameters: fragmentSize=1, summits=0. ATAC peaks with FDR <0.05 were considered as significantly differentially accessible regions and used for generating a volcano plot and heatmap comparing Sensitized and Resolved samples. Signal profiling of these ATAC peaks (FDR <0.05) along with their neighboring regions were performed using deeptools v3.3.0. Functional annotation of peaks (GO biological process) and peak-gene association were done with GREAT using the default "basal plus extension" parameter.

WGBS libraries were sequenced on an NovaSeq S4 300XP machine, with a total of ~300 million reads, by MGI institute.
FastqQC v0.11.8 was used to assess the quality of the raw reads. Following, the paired end reads were trimmed to remove adapter sequences and low quality reads with Cutadapt v1.18 using the parameters ("-q 15,10 -u 10 -U 15 --minimum-length 36") and reassessed using FastqQC. The mouse reference genome mm10 was first converted into a bisulfite converted version using Bismark v0.20.0 (bismark_genome_preparation). The paired end reads were aligned to the mm10 bisulfite converted genome using the options: "-N 1 -L 28 --score_min L,0,-0.6" and deduplicated using the deduplicate_bismark command from Bismark. Bismark command bismark_methylation_extractor was used to calculate DNA methylation levels and displayed in a methylC format showing coverage and CpG methylation percentage on the WashU Epigenome Browser. Bisulfite conversion was estimated using the conversion rate of cytosine to thymine in the lambda reference genome.
DMRs were identified with DSS v2.43.2 using the CpG methylation with coverage and methylation counts and calling DMRs between groups using the two-group comparison for biological replicates. The DMRs were called using the following parameters: DMLtest: "smoothing =TRUE, smoothing.span=500" and callDMR: "delta=0.2, p.threshold=0.001, minlen=200, minCG=3, dis.merge=50, pct.sig=0.5". PCA and Pearson correlation plots of the CpGs within DMRs using Deeptools v3.3.0. Biological replicates were combined by merging the paired fastq files between replicates and reprocessing the combined fastqs using the steps previously described. CpG density was visualized using a 5x coverage cutoff and ggplot2-v3.3.6 density function.
Sensitized-specific DMRs were defined as the overlapping regions between Naïve vs Sensitized and Resolved vs Sensitized DMRs comparisons. The percent methylation for sensitized-specific DMRs were visualized using R package "ComplexHeatmap" and DMRs were clustered using the Pearson method. DMRs with missing CpG values in any of the samples were removed. The top node defines two clusters: hypo and hyper Sensitized DMRs. The DNA methylation over Sensitized-specific hypo-DMRs were plotted using Deeptools plotProfile command and visualized using ggplot2 smooth function. Overlapping regions between DMR comparisons was performed and visualized by Intervene Venn function using default parameters. GREAT analysis was performed on Sensitized hypo-DMR cluster using the default "basal plus extension" parameter and the top 15 enriched pathways plotted. The Sensitized hypo-DMR cluster was also analyzed for genomic annotation using UCSC table browser (https://genome.ucsc.edu/cgi-bin/hgTables) to download GENCODE M25 annotation (https://www.gencodegenes.org/mouse/release_M25.html). Exon was defined at 5′ UTR, coding exon and 3′ UTR, and promoter as 1 kb upstream of TSS. DMRs were assigned to annotation if the DMR overlapped the 20% of the annotation using BEDTools intersect v2.27.1. Genomic annotation priority was assigned in the following order: Promoter, Coding Exon, 5′ UTR, 3′ UTR, intron and intergenic. DMRs assigned to promoters were used to plot DNA methylation change between Sensitized and Resolved against the log2 fold change of associated genes between Sensitized and Resolved differentiated urothelia treated with PBS and infection (UTI89).

For CUT&RUN data analysis, FastqQC v0.11.9 was used to assess the quality of the raw reads. Following, the paired end reads were trimmed with Cutadapt v1.9 to remove adapter sequences and low-quality reads with options: "--quality-cutoff=15,10 --minimum-length=36 -u 10 -U 10" and reassessed using FastqQC. Paired reads were then aligned using bowtie2 v2.3.4.1 with options: "–local–very-sensitive-local–no-unal–no-mixed–no-discordant–phred33 -I 10 -X 700". Mitochondrial reads were removed using samtools v1.9 and deduplicated using Picard v2.8.1 MarkDuplicates command. Properly paired and uniquely mapped reads were extracted using samtools view with options: -"h -b -q 10 -f 2". Peaks were called using MACS2 v2.1.1.20160309 callpeak with options: "-f BAMPE -q 0.01 --keep-dup all" for narrow peaks, H3K4Me3 and H3K27Ac and options: "-f BAMPE -q 0.05 --broad --keep-dup all" for broad peak, H3K27Me3. Encode defined blacklisted regions were removed. For each histone modification, a consensus peak list was used to calculate the fraction of reads in peaks (FRIP). The properly paired and uniquely mapped reads were converted to bigWig format using using Deeptools command bamCoverage and normalizing to read coverage and inverse FRIP score with options: "--binSize 10 --normalizeUsing RPGC –scaleFactor 1/FRIP". The normalized biological replicates were combined using ucsc-bigwigmerge v377 and converted from bedGraph to bigWigs using kentUCS v334 bedGraphToBigWig and mm10 chromosome sizes from UCSC (http://hgdownload.cse.ucsc.edu/goldenPath/mm10/bigZips/mm10.chrom.sizes). To profile Sensitized-specific DMR regions for other epigenetic modifications, the Sensitized hypo-DMRs were overlapped with MASC2 narrow peak regions for ATAC, H3K4Me3 and H3K27Ac and broad peak for H3K27Me3. The corresponding peak score was normalized using the inverse FRIP score and plotted using R package "ComplexHeatmap". Using the normalized bigWig tracks, the ATAC, H3K4Me3, H3K27Ac, H3K27Me3 signals were plotted over the Sensitized-specific hypo-DMRs using Deeptools plotProfile. The normalized bigWig signals were used for the casp1 heatmap.

For manuscripts utilizing custom algorithms or software that are central to the research but not yet described in published literature, software must be made available to editors and reviewers. We strongly encourage code deposition in a community repository (e.g. GitHub). See the Nature Portfolio guidelines for submitting code & software for further information.

Data

Policy information about availability of data

All manuscripts must include a data availability statement. This statement should provide the following information, where applicable:

- Accession codes, unique identifiers, or web links for publicly available datasets
- A description of any restrictions on data availability
- For clinical datasets or third party data, please ensure that the statement adheres to our policy

The authors declare that the data supporting the findings of this study are available within the paper, Supplementary Information, or Source Data. RNA-seq, ATAC-seq, WGBS, and CUT&RUN data have been deposited at NCBI under BioProject ID no. PRJNA705407. WashU Epigenome Browser map visualizing ATAC-seq, WGBS-seq, and CUT&RUN, and RNA-seq (forward-strand:green and reverse-strand:orange) data is accessible with the links:
Combined replicates:
https://epigenomegateway.wustl.edu/browser/?genome=mm10&noDefaultTracks=1&hub=https://wangftp.wustl.edu/~jharrison/PUBLISHED_DATAHUBS/Hultgren/Russell_Bacterial_infection_combined.json
Combined and individual replicates:

https://epigenomegateway.wustl.edu/browser/?genome=mm10&noDefaultTracks=1&hub=https://wangftp.wustl.edu/~jharrison/PUBLISHED_DATAHUBS/Hultgren/Russell_Bacterial_infection_all.json

Field-specific reporting

Please select the one below that is the best fit for your research. If you are not sure, read the appropriate sections before making your selection.

☒ Life sciences ☐ Behavioural & social sciences ☐ Ecological, evolutionary & environmental sciences

For a reference copy of the document with all sections, see nature.com/documents/nr-reporting-summary-flat.pdf

Life sciences study design

All studies must disclose on these points even when the disclosure is negative.

Sample size	No statistical methods were used to predetermine sample size for experiments in this study. Experiments were performed at least in triplicate for non-mouse experiments. A minimum of n=3 was chosen as a sample size to generate robust and reliable results on the cell lines based on similar studies in the field. For mouse infection experiments, sample sizes were determined based on previous experience with the same recurrent infection model studies (e.g. O'Brien et al., Nat Microbiol, 2016).
Data exclusions	No exclusion was applied to the uploaded raw data deposited at NCBI. Data for the R1 and S2 cell lines was excluded from the cell size measurement (Fig. 2f-g) and RNA-seq analysis (Fig. 3g-h) experiments, respectively, due to the development of phenotypic changes (poor terminal differentiation) that arose with later passage and were likely the result of spontaneous mutation. We subsequently confirmed that cultures from earlier passages of these two cell lines lack these phenotypic changes. For ATAC-seq, we selected in an unbiased manner the best two biological replicates for each disease history among raw data based on sequencing quality control. Then these two cell lines were used for further WGBS and CUT&RUN analyses.
Replication	We have established and characterized four cell lines isolated from Naive, Resolved, and Sensitized mice. Each cell line is a biological replicate and different culture can be a biological or a technical replicate depending on how you define. RNA-seq and ATAC-seq data showed they are well grouped by infection history. All technical and biological replications of experiments were successful. For mouse secondary infection, 2-3 independent experiments were conducted.
Randomization	Randomization was not relevant to this study as the samples were treated similarly. For mouse infection, mice with an initial bacterial infection were defined as Resolved or Sensitized mice based on their infection outcomes and age-matched Naive mice with mock-infection were used as control. Then these Naive, Resolved and Sensitized mice were challenge infected using the same infection protocol. For primary USC lines, Naive, Resolved and Sensitized USC cultures were treated similarly for each experiment.
Blinding	Blinding is not relevant to our study as all samples were treated similarly. Blinding was also not possible as the same person designed, performed and analyzed the experiments.<br>Exceptionally, confocal imaging of Naive, Resolved and Sensitized differentiated urothelia and the measurement of surface cell size was conducted in a double-blind manner to avoid biased selection of representing cell surface area for microscopic imaging. The other in vivo and in vitro experiments didn't have concern of biased selection, so blinding was not relevant.

Reporting for specific materials, systems and methods

We require information from authors about some types of materials, experimental systems and methods used in many studies. Here, indicate whether each material, system or method listed is relevant to your study. If you are not sure if a list item applies to your research, read the appropriate section before selecting a response.

Materials & experimental systems

n/a	Involved in the study	
☐	☒	Antibodies
☐	☒	Eukaryotic cell lines
☒	☐	Palaeontology and archaeology
☐	☒	Animals and other organisms
☒	☐	Human research participants
☒	☐	Clinical data
☒	☐	Dual use research of concern

Methods

n/a	Involved in the study	
☒	☐	ChIP-seq
☒	☐	Flow cytometry
☒	☐	MRI-based neuroimaging

Antibodies

Antibodies used	Mouse Monoclonal anti-Caspase-1 (p20) (Casper-1), Adipogen, cat. AG-20B-0042-C100, 1 mg/ml (1:5,000)<br>2nd: Anti-mouse IgG, HRP-linked antibody, Cell Signaling Technology, cat #7076S, 2 mg/ml (1:3,000)<br>Beta Actin Loading Control Monoclonal antibody (BA3R), Invitrogen, cat. MA5-15739, 1 mg/ml (1:10,000)

2nd: Anti-mouse IgG, HRP-linked antibody, Cell Signaling Technology, cat #7076S, 2 mg/ml (1:3,000)

Alexa Fluor 555 Phalloidin, ThermoFisher Scientific, cat. A34055, 300 units (1:200)
4′,6-diamidino-2-phenylindole (DAPI), ThermoFisher Scientific, cat. D1306 (1:1,000)

Mouse monoclonal anti-keratin 20 (K20), Ks20.8, Abcam, cat. ab854 (Lot #GR292727-7) (1:200)
2nd: Alexa Fluor 647 Donkey anti-mouse IgG (H+L), Invitrogen, cat. A-31571, 2 mg/ml (1:1,000)
Goat Polyclonal anti-E-cadherine (Ecad), R&D Systems, cat. AF748 (Lot #CYG0516081), 0.2 mg/ml (1:500)
2nd: Alexa Fluor 568 donkey anti-goat IgG (H+L), Abcam, cat. ab175704, 2 mg/ml (1:1,000)
Goat polyclonal anti-Uroplakin 3a (Upk3a), M-17, Santa Cruz Biotechnology, cat. sc-15186, 0.2 mg/ml (1:500)
2nd: Alexa Fluor 647 donkey anti-goat IgG (H+L), Invitrogen, cat. A32849, 2 mg/ml (1:1,000)
Rabbit polyclonal anti-p63 (N2C1), Internal, GeneTex, cat. GTX102425 (Lot #43138), 0.34 mg/ml (1:1,000)
2nd: Alexa Fluor 555 donkey anti-rabbit IgG (H+L), Abcam, cat. ab150074, 2 mg/ml (1:1,000)
Rabbit monoclonal anti-Keratin 5 (K5) (EP1601Y), Abcam, cat. ab52635 (Lot #GR3292032-14), 0.126 mg/ml (1:100)
2nd: Alexa Fluor 555 donkey anti-rabbit IgG (H+L), Abcam, cat. ab150074, 2 mg/ml (1:1,000)
Mouse monoclonal anti-keratin 14 (K14) (LL001), Santa Cruz Biotechnology, cat. sc-53253 (Lot # C3021), 0.2 mg/ml (1:50)
2nd: Alexa Fluor 488 donkey anti-mouse IgG IgG (H+L), Invitrogen, cat. A-21202, 2 mg/ml (1:1,000)
Mouse Monoclonal anti-Uroplakin 3a (Upk3a) (AU1), Fitzgerald, cat. 10R-U103a, 50 ug/ml (1:50)
2nd: Alexa Fluor 647 Donkey anti-mouse IgG (H+L), Invitrogen, cat. A-31571, 2 mg/ml (1:1,000)

Rabbit monoclonal anti-H3K4Me3 (C42D8), Cell Signaling Technology, cat. 9751, 100 µg/ml (1:50)
Rabbit monoclonal anti-H3K27A (D5E4), Cell Signaling Technology, cat. 8173, 100 µg/ml (1:100)
Rabbit monoclonal anti-H3K27Me3 (C36B11), Cell Signaling Technology, cat. 9733, 100 µg/ml (1:50)

Validation

anti-Caspase-1 (p20): This antibody recognizes endogenous full-length and activated (p20 fragment) mouse caspase-1. Described to cross-react with full-length and activated (p20 fragment) of rat caspase-1 (Lit. #30 & 34). This antibody is purified from concentrated hybridoma tissue culture supernatant. Crossreactivity is for Mouse and Rat, and applications is verified for WB, IHC (P), and IP.

Beta Actin Loading Control Monoclonal Antibody (BA3R): Reactivity has been confirmed on western blots with whole cell extracts of different cell lines and tissue lysates from chicken, human, mouse, rat, and rabbit, and identifies the target band at 42 kDa. Images shown on the website. Published species are Chicken, Fungi, Guinea pig, Human, Mouse, Non-human primate, Pig, Plant, Rabbit, and Rat. Available applications are Wb, IHC, IHC(P), ICC/IF, Flow Cytometry, ELISA, IP.

Alexa Fluor 555 Phalloidin: Phalloidin is a bicyclic peptide belonging to a family of toxins isolated from the deadly Amanita phalloides 'death cap' mushroom and is commonly used in imaging applications to selectively label F-actin. Phalloidin binds F-actin with high selectivity while Alexa Fluor® 555 provides red-orange fluorescence of unparalleled brightness and photostability. Staining was validated by using A549, BPAE, HCASM, HeLa, and U2OS cell lines.

DAPI: A popular nuclear and chromosome counterstain, DAPI emits blue fluorescence upon binding to AT regions of DNA. Although the dye is cell impermeant, higher concentrations will enter a live cell. Application for cell imaging with fluorescence microscope is verified on the website.

anti-K20: This antibody is highly specific to cytokeratin 20 and shows no cross-reaction with other intermediate filament proteins. It is essentially non-reactive in squamous cell carcinomas and adenocarcinomas of the breast, lung, and endometrium, non-mucinous tumors of the ovary and small cell carcinomas. Immunogen is electrophoretically purified Keratin K20 from Human intestinal mucosa. Tested applications are Flow Cyt, IHC-P, ICC/IF.

anti-Ecad: Immunogenicity is mouse myeloma cell line NS0-derived recombinant mouse E-Cadherin Asp157-Val709 (Accession # P09803). The manufacturer validated reactivity to human and mouse by CyTOF-ready, Flow Cytometry, Immunocytochemistry/ Immunofluorescence, Immunohistochemistry, Simple Western, and Western Blot. Data on website.
Product citation: O'Brien, V. P. et al. Nat Microbiol 2, 16196, doi:10.1038/nmicrobiol.2016.196 (2016).

anti-Upk3a (M-17): UPIIIa (M-17) is recommended for detection of UPIIIa of mouse, rat and human origin by immunofluorescence (starting dilution 1:50, dilution range 1:50-1:500). UPIIIa (M-17) is also recommended for detection of UPIIIa in additional species, including equine, canine and porcine. Molecular Weight of UPIIIa: 47 kDa. Positive Controls: mouse bladder extract: sc-364919.
Product citations: Nagamatsu, K. et al. Proc Natl Acad Sci U S A 112, E871-880 (2015).

anti-p63: This antibody was validated by the manufacturer using human small cell lung cancer (SCLC) and non-small cell lung cancer (NSCLC) specimens. Antibody detects p63 protein at nucleus by immunohistochemical analysis using paraffin-embedded mouse tongue. This antibody was verified reactivity for mouse and rat, and predicted reacvitiy is for Zebrafish, Bovine, Chicken. Available applications are Wb, ICC/IF, IHC(P), IHC-Fr, IP, Proximity ligation assay.

anti-K5: This monospecific polyclonal antibody was raised against a peptide sequence derived from the C-terminus of the mouse keratin 5 protein. Each lot of this antibody is quality control tested by the manufacturer by immunohistochemical staining of human skin and mouse skin. This antibody has reactivity with Mouse, Rat, Human and suitable for Flow Cytometry, ICC/IF, WB, IHC (P).

Anti-K14(LL001): This antibody is mouse monoclonal Cytokeratin 14 antibody (also designated Cytokeratin 14 antibody or KRT14 antibody) suitable for the detection of Cytokeratin 14 of mouse, rat and human origin by WB, IP, IF and IHC(P). It is available conjugated to Alexa Fluor® 488, Alexa Fluor® 546, Alexa Fluor® 594 or Alexa Fluor® 647 for WB (RGB), IF, IHC(P) and FCM, and for use with RGB fluorescent imaging systems

anti-Upk3a (AU1): Uroplakin III contributes in constituting the asymmetrical unit membrane of the plaques of urothelial superficial (umbrella) cells. Uroplakin is a membrane glycoprotein (47 kDa) and has been shown to be a specific marker of terminal urothelial differentiation. Uroplakin III antibody was raised in mouse using AUM preparation from bovine urinary bladder as the immunogen.
Product citation: O'Brien, V. P. et al. Nat Microbiol 2, 16196, doi:10.1038/nmicrobiol.2016.196 (2016).

anti-H3K4Me3 (C42D8): This antibody detects endogenous levels of histone H3 when tri-methylated on Lys4. This antibody shows some cross-reactivity with histone H3 that is di-methylated on Lys4, but does not cross-react with non-methylated or mono-methylated histone H3 Lys4. In addition, the antibody does not cross-react with methylated histone H3 Lys9, Lys27, Lys36 or methylated histone H4 Lys20. Species reactivity was confirmed for Human, Mouse, Rat, Monkey, D. melanogaster, S. cerevisiae. Available application is WB, IF, IHC (P), Flow Cytometry, Chromatin IP, Chromatin IP-seq, and CUT&RUN.

anti-H3K27A (D5E4): This antibody recognizes endogenous levels of histone H3 protein only when acetylated at Lys27. This antibody does not cross react with histone H3 acetylated at Lys9, 14, 18, 23, or 56. This antibody shows some cross-reactivity with acetyl-histone H2B lysine 5. Species reactivity is for Human, Mouse, Rat, Monkey and available applications are WB, IF, Flow Cytometry, Chromatin IP, Chromatin IP-seq, CUT&RUN.

anti-H3K27Me3 (C36B11): This antibody detects endogenous levels of histone H3 only when tri-methylated on Lys27. The antibody does not cross-react with non-methylated, mono-methylated or di-methylated Lys27. In addition, the antibody does not cross-react with mono-methylated, di-methylated or tri-methylated histone H3 at Lys4, Lys9, Lys36 or Histone H4 at Lys20. Species reactivity is confirmed for Human, Mouse, Rat, Monkey and available applications are WB, IHC Leica Bond, IHC (P), IF, Flow Cytometry, Chromatin IP, Chromatin IP-seq, CUT&RUN.

Eukaryotic cell lines

Policy information about cell lines

Cell line source(s)

Human BECs, designated 5637 (ATCC HTB-9) cells, were obtained from the American Type Culture Collection.

Primary bladder epithelial cell lines: Juvenile naive, Naive, Resolved, and Sensitized urothelial stem cells (USCs) were isolated from each 8 weeks old female Juvenile naive C3H/HeN mice and adult female Naive, Resolved, and Sensitized C3H/HeN mice, respectively. USCs were cultured and passaged for at least 10 passages before use in experiments to avoid remaining non-stem urothelial cells.

Authentication

No authentication was performed.

Mycoplasma contamination

5637 cells, Juvenile, Naive, Resolved and Sensitized USC lines were tested negative for mycoplasma contamination by PCR.

Commonly misidentified lines
(See ICLAC register)

No commonly misidentified cell lines were used.

Animals and other organisms

Policy information about studies involving animals; ARRIVE guidelines recommended for reporting animal research

Laboratory animals

Female C3H/HeN mice were obtained from Envigo (Indianapolis, IN). All mice were seven to eight weeks old ('juvenile') at the time of the initial infection. For mouse housing condition, the 12 light /12 dark cycle is used. Temperature is 72°F and humidity range is 30 - 70% are maintained in the facility.

Wild animals

The study did not involve wild animals.

Field-collected samples

The study did not involve samples collected from the field.

Ethics oversight

All animal experimentation was conducted according to the National Institutes of Health guidelines for the housing and care of laboratory animals. All experiments were performed in accordance with institutional regulations after review and approval by the Animal Studies Committee at Washington University School of Medicine in St Louis, Missouri.

Note that full information on the approval of the study protocol must also be provided in the manuscript.
