# Supplementary figures and images for: Uropathogenic Escherichia coli infection-induced epithelial trained immunity impacts urinary tract disease outcome
Source: Nat Microbiol. 2023 Apr 10;8(5):875–88. doi: 10.1038/s41564-023-01346-6 (PMC10159856; doi:10.1038/s41564-023-01346-6)

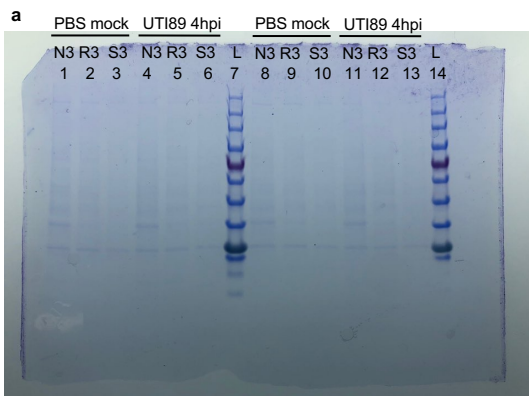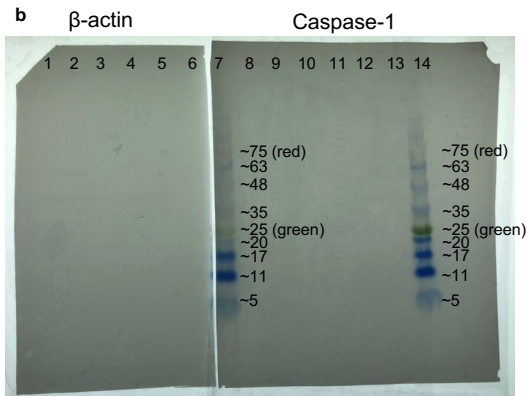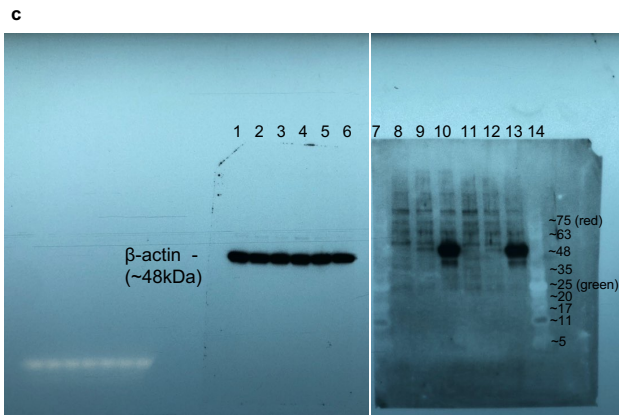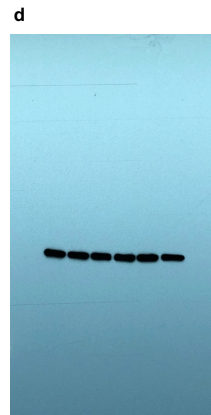

Supplement: Source Data Fig. 5 — Fig. 5e. Uncropped gel and blot images of N3, R3 and S3 differentiated urothelia. a, Whole gel loaded with two sets of 6 samples (PBS mock-infected N3, R3 and S3 urothelial cells and UTI89-infected N3, R3 and S3 urothelial cells) with Tris-glycine 4–20% loading dye. Both uninfected and infected sensitized urothelial cells had caspase-1 staining. Only mock-infected N3, R3 and S3 data were used for the paper since sensitized cells expressed caspase-1 regardless of infection condition. b, Membrane image (1–6 lane cut was used for β-actin staining and 7–14 lane cut was used for caspase-1 staining). c, Film development was done separately since β-actin and caspase-1 staining needed different exposure times (1 min and 3 min, respectively). β-actin (~48 kDa) expression was similar among all the samples, while only sensitized cells had staining at P45 pro-caspase-1 (~45 kDa) and P35 caspase-1 (~35 kDa) locations. For Fig. 5e in the manuscript, (d) 20 s exposure image was used instead of 1 min exposure for β-actin for cleaner band separation. [file 41564_2023_1346_MOESM8_ESM.pdf]
